# Public Perceptions of Physician Attire and Professionalism in the US

**DOI:** 10.1001/jamanetworkopen.2021.17779

**Published:** 2021-07-30

**Authors:** Helen Xun, Jonlin Chen, Alexander H. Sun, Hillary E. Jenny, Fan Liang, Jordan P. Steinberg

**Affiliations:** 1 Department of Plastic & Reconstructive Surgery, Johns Hopkins University School of Medicine, Baltimore, Maryland; 2 Division of Plastic Surgery, R. Adams Cowley Shock Trauma Center, University of Maryland School of Medicine, Baltimore

## Abstract

**Importance:**

In recent years, casual physician attire (fleece jackets and softshell jackets) has become increasingly popular, but to our knowledge, public perceptions of these garments have not been studied. Furthermore, gender biases may result in differing expectations and perceptions of female and male physicians and may be associated with patient rapport and trust building.

**Objective:**

To characterize public perceptions of casual physician attire and implicit gender biases in public assessment of physicians’ professional attire.

**Design, Setting, and Participants:**

This survey study used a population-based survey administered via Amazon Mechanical Turk from May to June 2020 among individuals aged 18 years or older who were US residents and for whom English was the primary language.

**Intervention:**

Survey featuring photographs of a male or female model wearing various types of physician attire (white coat, business attire, and scrubs).

**Main Outcomes and Measures:**

Respondents’ ratings of professionalism, experience, and friendliness of the male and female models in various attire and perceptions of the models’ most likely health care profession. Preference scores for various outfits were calculated as the difference between the preference score for an outfit and the mean preference score for the outfit-role pairing.

**Results:**

Of 522 surveys completed, 487 were included for analysis; the mean (SD) age of respondents was 36.2 (12.4) years, 260 (53.4%) were female, and 372 (76.4%) were White individuals. Respondents perceived models of health care professionals wearing white coats vs those wearing fleece or softshell jackets as significantly more experienced (mean [SD] experience score: white coat, 4.9 [1.5]; fleece, 3.1 [1.5]; softshell, 3.1 [1.5]; *P* < .001) and professional (mean [SD] professionalism score: white coat, 4.9 [1.6]; fleece, 3.2 [1.5]; softshell, 3.3 [1.5]; *P* < .001). A white coat with scrubs attire was most preferred for surgeons (mean [SD] preference index: 1.3 [2.3]), whereas a white coat with business attire was preferred for family physicians and dermatologists (mean [SD] preference indexes, 1.6 [2.3] and 1.2 [2.3], respectively; *P* < .001). Regardless of outerwear, female models in business attire as inner wear were rated as less professional than male counterparts (mean [SD] professionalism score: male, 65.8 [25.4]; female, 56.2 [20.2]; *P* < .001). Both the male and the female model were identified by the greater number of respondents as a physician or surgeon; however, the female model vs the male model was mistaken by more respondents as a medical technician (39 [8.0] vs 16 [3.3%]; *P* < .005), physician assistant (56 [11.5%] vs 11 [2.3%]; *P* < .001), or nurse (161 [33.1%] vs 133 [27.3%]; *P* = .050).

**Conclusions and Relevance:**

In this survey study, survey respondents rated physicians wearing casual attire as less professional and experienced than those wearing a white coat. Gender biases were found in impressions of professionalism, with female physicians’ roles being more frequently misidentified. Understanding disparate public perceptions of physician apparel may inform interventions to address professional role confusion and cumulative career disadvantages for women in medicine.

## Key Points

**Question:**

How does the public perceive casual physician attire compared with white coats, and are there differences by gender of the physician?

**Findings:**

In this survey study of 487 survey respondents, physicians wearing white coats were perceived as significantly more experienced, professional, and friendly compared with those wearing fleece or softshell jackets. Photographed female models were rated as appearing less professional than the male models and were more likely to be mistaken as a medical technician, physician assistant, or nurse.

**Meaning:**

The findings suggest that individuals prefer that physicians wear white coats and that gender biases in the perception of professional physician attire exist.

## Teaser

This survey study uses data from a national survey of US adults to investigate perceptions of physician professionalism based on attire and whether gender biases exist.

## Introduction

The 19th-century advent of antisepsis and evidence-guided medical practices^1^ contributed to the emergence of the physician’s white coat as a symbol of cleanliness, scientific achievement, and professional responsibility.^2^ This symbolism has persisted into the 21st century; physicians who wear white coats are viewed as more knowledgeable and competent and more likely to build rapport with patients.^3,4,5^ The majority of patients prefer formal physician attire, and more than one-third of patients agree it is a component of satisfaction with care.^6^ Preference for particular attire may be associated with patient age, location, culture, specialty of the physician, and context of care.^3,6,7^

Despite its longstanding history, the white coat has come under some scrutiny, including its purported association with nosocomial transmission of pathogens and allergens.^8,9,10,11,12^ This has led to institutional laundering recommendations^13^ and policies requiring that the lower arms and wrists be exposed.^14^ Moreover, some medical specialties find it more practical not to wear the white coat, particularly surgeons who find scrubs to be more pragmatic attire for transitioning between operating rooms, the clinic, and the hospital floor.^15^ The white coat was traditionally also worn for identification to reinforce hierarchical delineation.^16^ As the health care workplace transforms into a team-based environment^17^ with emphasis on patient safety rather than hierarchy, the white coat has appeared to some as an antiquated relic.^18^

The white coat has also brought to the forefront gender biases that female physicians may experience. Rehman and colleagues^19^ found in 2005 that a female physician’s attire was associated with trust and confidence building more often than was a male physician’s attire. Female physicians are also held to higher sartorial standards; in 1 study,^20^ 73% of patients voted that business attire without a white coat was inappropriate for female physicians compared with 24% for male physicians. Collectively, these differences in the public’s expectations of physicians on the basis of clothing and gender reflect an embedded pattern of gender bias, ultimately affecting physician selection and patient-physician relationships.^21^

In recent years, casual physician attire has gained popularity as an alternative to white coats. Casual physician attire includes fleece and softshell jackets or vests and may feature institutional insignia and/or identification of the wearer. Fleece jackets are characterized by a polyester knit advertised as a wool-like aesthetic,^22^ whereas softshell jackets are typically a polyester blend with polyurethane coating, advertised to be windproof and waterproof.^23^ This casual wear is either individually purchased or acquired in bulk by groups or institutions.^24^ Often, practitioners may wear the casual attire as a more modern alternative in place of the white coat owing to comfort, ease of transition to and from work, and/or practical reasons such as warmth or weatherproof function.^18^

Despite the popularity of physician casual wear, this emerging trend has not been well studied, nor have the issues of institutional oversight or professional societies’ guidelines for these garments. Therefore, the first objective of this study was to characterize public perceptions of casual physician attire, including fleece and softshell jackets, compared with the traditional professional physician attire of business suits and a white coat. In addition, the loss of the identifying white coat, coupled with entrenched gender stereotypes in medicine, has blurred the lines of role identification and hierarchical delineation.^5^ Although mistaken professional roles based on gender stereotypes are a pervasive phenomenon that has been anecdotally described,^25,26^ few reports have focused on the specific analysis of implicit gender bias and its consequences.^27,28^ Thus, a second objective of this study was to characterize implicit gender biases and how they may influence explicit practices such as role identification and physician impressions in the health care setting. We hypothesized that the public would prefer traditional professional physician attire compared with casual attire and that despite new physician attire, gender disparities would persist.

## Methods

This survey study was reviewed and approved by the Institutional Review Board of Johns Hopkins University, and participants were informed that completion of the survey served as consent to participate. The study followed the Strengthening the Reporting of Observational Studies in Epidemiology (STROBE) reporting guideline for cross-sectional studies.

### Survey Instrument

Our study team developed a web-based survey using Qualtrics software and administered the survey through Amazon Mechanical Turk (MTurk) from May to June 2020 (eAppendix 1 in the Supplement). The survey was first pilot tested in a cohort of 100 MTurk respondents, and survey responses were verified before the survey was distributed to a larger sample. Survey respondents were restricted to individuals aged 18 years or older who were US residents and identified English as their primary language. Respondents who successfully completed all survey questions received $0.25. Surveys were excluded if attention-check questions were incorrect. Demographic information, prior exposure to health care, and the health care employment status of respondents or their family members were self-reported.

### Assessment of Casual Physician Attire

Respondents were asked to characterize on a 5-point Likert scale how often and in which locations they see health care professionals wearing a white coat, scrubs, fleece-blended sweaters or vests, and softshell jackets (1 indicated “always” and 5 “never”). Respondents ranked what was most important in seeking a health care professional: experience, professionalism, or friendliness. Respondents were then presented with a series of photographs of deidentified models wearing permutations of various health care attire and asked to rank the attire on a 6-point Likert scale, with 1 indicating least experienced, professional, and friendly and 6 indicating most experienced, professional, and friendly. In addition, respondents were asked to rank their preference for health care attire based on a given professional’s role (ie, “A nurse walks into your patient room. What would you prefer to see him or her wear?”). Health care roles surveyed included nurse, technician, phlebotomist, family physician, dermatologist, and surgeon; health care roles were selected based on prior literature reports establishing precedent.^4,19,29^

### Assessment of Professionalism and Role by Gender

**Figure 1.**
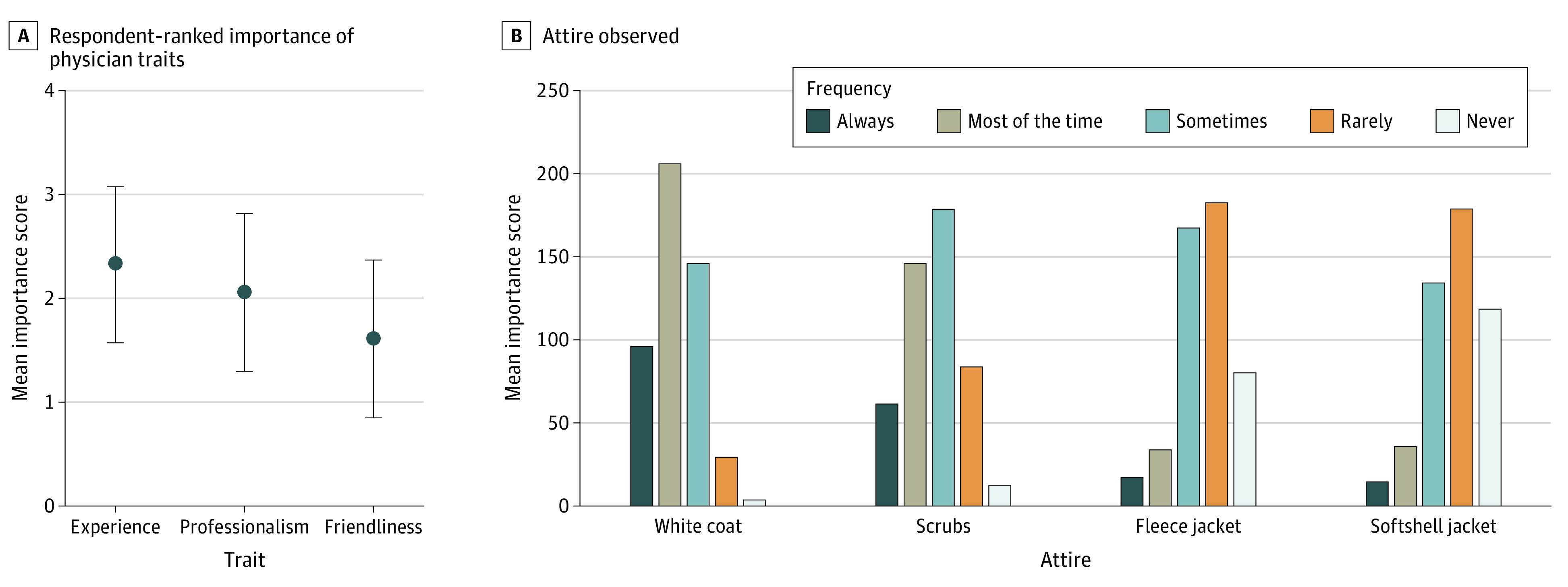
Preferred Traits of and Prevalence of Attire Worn by Health Care Professionals A, Dots indicate mean values; whiskers, SDs. *P* < .001 for all comparisons. B, Respondents were shown photographs of attire and asked how often they had seen health care professionals in that attire. Scrubs refer to unisex, hospital-grade scrubs.

Study respondents were presented with a series of photographs of deidentified models wearing several permutations of nonbranded health care attire consisting of inner wear (business attire or scrubs) with or without outerwear (long white coat, fleece jacket, or softshell jacket) (Figure 1A). Participants were presented with a series of randomized individual images of either a male or female model in each attire permutation and were asked to rate the professionalism of the model from 0 to 100, with 100 indicating “most professional.” Participants were then asked to identify the profession of a male and a female model as physician, surgeon, nurse, medical technician, or physician assistant.

### Statistical Analysis

All variables were analyzed using GraphPad Prism statistical software, version 6.04 (GraphPad Software). Ranks and preferences for the physician were converted to a numeric, inverse score such that the characteristic with the highest rank (1) resulted in the highest score (3 or 6). Preference scores for various outfits were calculated as the difference between the preference score for the outfit and the mean preference score for the outfit-role pairing. For example, for a white coat–surgeon pair with a respondent score of 3 and a mean preference score of 4.5, the calculated preference score was −1.5. Experience, professionalism, and friendliness scores and preference indexes were compared using the nonparametric Friedman test and the Dunn multiple comparisons test after Shapiro-Wilk normality testing, with adjustment for multiple comparisons. Multivariate analysis was used to assess factors associated with experience, professionalism, and friendliness scores using Stata, version 15 (Stata Corp LLC). Factors analyzed included all respondent characteristics collected (age, gender, educational level, income, geographic location, health care location where respondents received most of their health care, and whether the respondent was or had a family member who was a health care worker). Statistical significance was set at *P* = .05 using a 2-tailed test. Post hoc power analyses were conducted for the nonparametric Friedman test, Dunn multiple comparisons test, and multivariate analysis, and only adequately powered statistical comparisons were made.

## Results

Of the 522 surveys administered and completed, 487 (93.3%) were included in the final analysis; 35 surveys were excluded owing to incorrect attention-check question responses. The mean (SD) age of respondents was 36.2 (12.4) years, 260 (53.4%) identified as female, 372 (76.4%) were White individuals, and 33 (6.8%) were Black or African American. Demographic data of included respondents are reported in Table 1. Respondents’ exposure to health care is shown in Table 2.

**Table 1.** Demographic Characteristics of Survey Respondents Included in Final Analysis

Characteristic	Respondents, No. (%) (N = 487)^a^
Age, mean (SD), y	36.2 (12.4)
Race/ethnicity	
White	372 (76.4)
Black or African American	33 (6.8)
American Indian or Alaska Native	15 (3.1)
Asian	71 (14.6)
Native Hawaiian or Pacific Islander	7 (1.4)
Other^b^	9 (1.8)
Prefer not to answer	1 (0.2)
Gender identity	
Male	222 (45.6)
Female	260 (53.4)
Nonbinary	4 (0.8)
Other^b^	1 (0.2)
Educational level	
Less than high school diploma	1 (0.2)
High school diploma or equivalent	28 (5.7)
Some college but no degree	73 (15.0)
Associate’s degree (2 y)	38 (7.8)
Bachelor’s degree (4 y)	243 (49.9)
Master’s degree	85 (17.5)
Doctoral or professional degree	19 (3.9)
Annual income, USD, thousands	
<25 to 50	121 (24.8)
51-75	113 (23.2)
76-100	96 (19.7)
101-150	57 (11.7)
151-200	17 (3.5)
>200	12 (2.5)
US region of residence^c^	
Northeast	80 (16.4)
West	127 (26.1)
Midwest	77 (15.8)
South	183 (37.6)

^a^ Data are presented as number (%) of participants unless otherwise indicated.

^b^ Other was an option provided for individuals who did not identify with any of the listed categories.

^c^ Categorized according to the US Census Bureau Regions and Divisions.

**Table 2.** Survey Respondents’ Exposure to Health Care

Exposure variable	Respondents (N = 487)^a^
Annual visits to health care practitioners, mean (SD), No.	
Family doctor or primary care physician	3.4 (2.5)
Internal medicine physician and specialists	2.4 (2.7)
Emergency department or urgent care	2.0 (2.5)
Dermatologist or cosmetic plastic surgery	1.8 (2.6)
Physical therapy or occupational therapy	2.0 (2.7)
Surgeon	1.7 (2.7)
Where majority of care is received	
Urban area	249 (51.1)
Suburban area	204 (41.9)
Rural community	34 (7.0)
Been admitted to the hospital	
Yes	292 (60.0)
No	195 (40.0)
Had surgery before	
Yes	260 (53.4)
No	227 (46.6)
Health care worker	
Yes	103 (21.1)
No	376 (77.2)
Not sure	8 (1.6)
If a health care worker, role in health care	
Practitioner	40 (8.2)
Administration	35 (7.2)
Support staff	28 (5.7)
Has a family member who works in health care	
Yes	154 (31.6)
No	324 (66.5)
Not sure	9 (1.8)
Family member’s role in health care	
Practitioner	56 (11.5)
Administration	49 (10.1)
Support staff	49 (10.1)

^a^ Data are presented as number (%) of participants unless otherwise indicated.

### Exposure to Health Care and Perceptions of Professional Attire

Experience was perceived as the most important trait of health care practitioners, followed by professionalism and friendliness (Figure 1A). Multivariate analyses of associations between respondent characteristics and preferred physician traits are provided in eAppendix 2 in the Supplement. Respondents most commonly reported seeing health care practitioners in white coats “most of the time” (208 respondents [42.5%]), in scrubs “sometimes” (179 [36.6%]), and in fleece and softshell jackets “rarely” (181 [37.0%]) (Figure 1B). The health care locations where respondents reported seeing various types of physician attire are shown in eTable 1 in the Supplement.

### Public Perceptions of Physician Traits and Their Association With Attire

**Figure 2.**
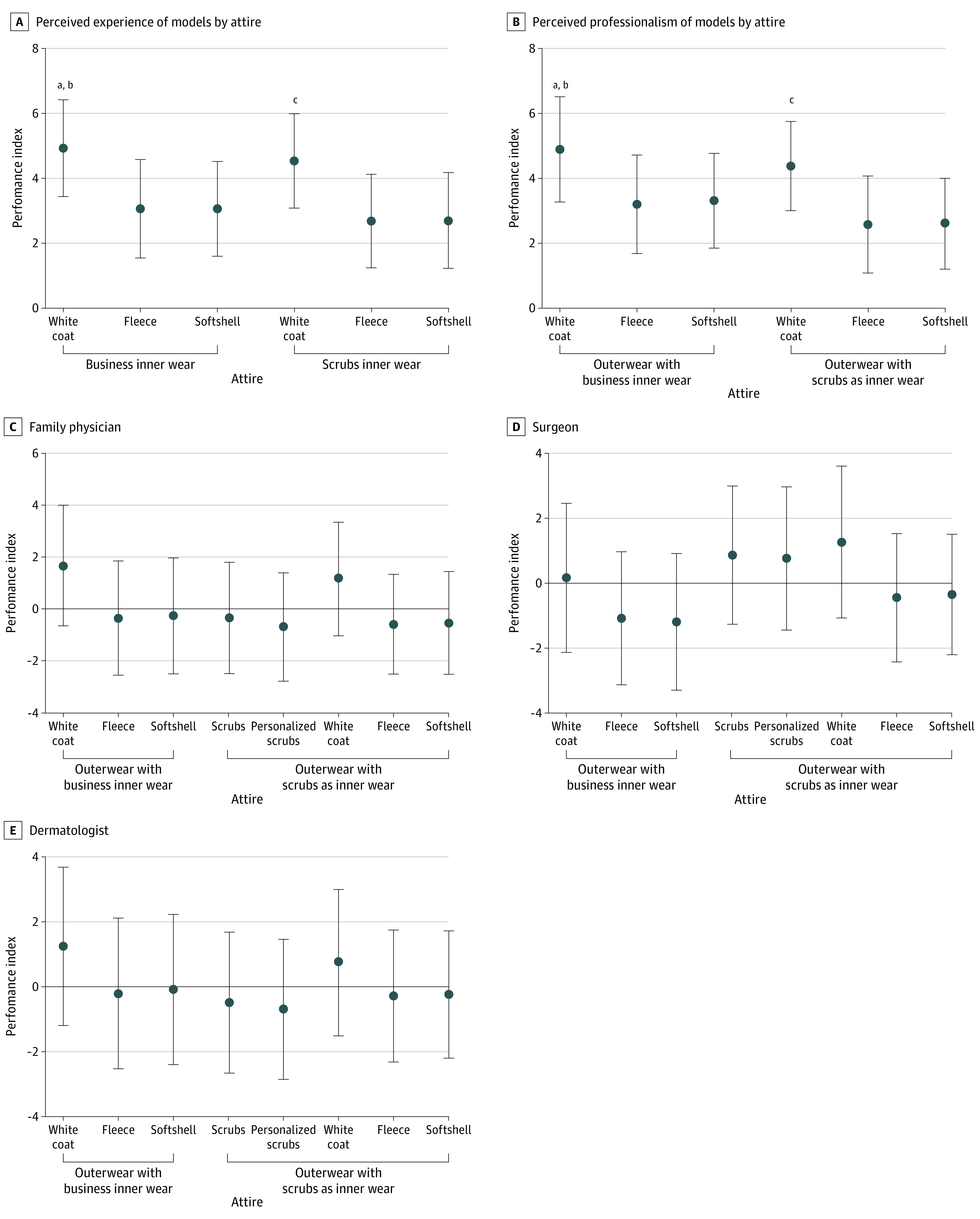
Respondent Ratings of Health Care Practitioners’ Professionalism and Experience by Attire and Preferred Attire by Type of Physician Scrubs refer to unisex, hospital-grade scrubs; personalized scrubs refer to branded and tailored scrubs, including different fits by gender. A and B, Respondents ranked physicians as most (6) to least (0) experienced or professional. C, Respondents were shown a model wearing business attire with varying outerwear, scrubs alone, or scrubs as inner wear with varying outerwear. Dots indicate mean values; whiskers, SDs. ^a^*P* < .001 for business inner wear with white coat vs with fleece or softshell. ^b^*P* = .02 for white coat with business inner wear vs with scrubs. ^c^*P* < .001 for scrubs with white coat vs with fleece or softshell.

For models shown in either business or scrub inner wear (Figure 2A), a white coat worn as outerwear, compared with a fleece jacket or a softshell jacket, was associated with perceptions of the practitioner being significantly more experienced (mean [SD] experience score: white coat, 4.9 [1.5]; fleece, 3.1 [1.5]; and softshell, 3.1 [1.5]; *P* < .001) and professional (mean [SD] professionalism score: white coat, 4.9 [1.6]; fleece, 3.2 [1.5]; softshell, 3.3 [1.5]*; P < *.001) (Figure 2B). A model in a white coat was seen as more friendly than a model in a softshell jacket (mean [SD] friendliness score: white coat, 3.6 [1.9]; softshell, 3.1 [1.6]; *P* < .003). Results of the multivariate analysis comparing experience, professionalism, and friendliness scores for physicians in various attire with background respondent characteristics are given in eAppendix 4 in the Supplement. Notable findings included a significant positive association between older respondent age and greater perceived experience of a model in a white coat with business inner wear (coefficient, 0.015; 95% CI, 0.004-0.026; *P* < .009). A fleece jacket worn with scrubs as inner wear was associated with decreased professionalism scores for all geographic regions in the US except the West.

### Attire Preference for Specific Health Care Roles

The distribution of attire preference indexes across health care roles is shown in Figure 2C and eAppendix 3 in the Supplement. Respondents preferred white coat outerwear with scrubs inner wear for surgeons (mean [SD] preference index: 1.3 [2.3]), whereas they preferred white coat outerwear with business inner wear for family physicians and dermatologists (mean [SD] preference indexes, 1.6 [2.3] and 1.2 [2.3], respectively; *P* < .001) (eAppendix 4 in the Supplement).

### Perceived Professionalism by Attire and Gender of Photograph Model

**Figure 3.**
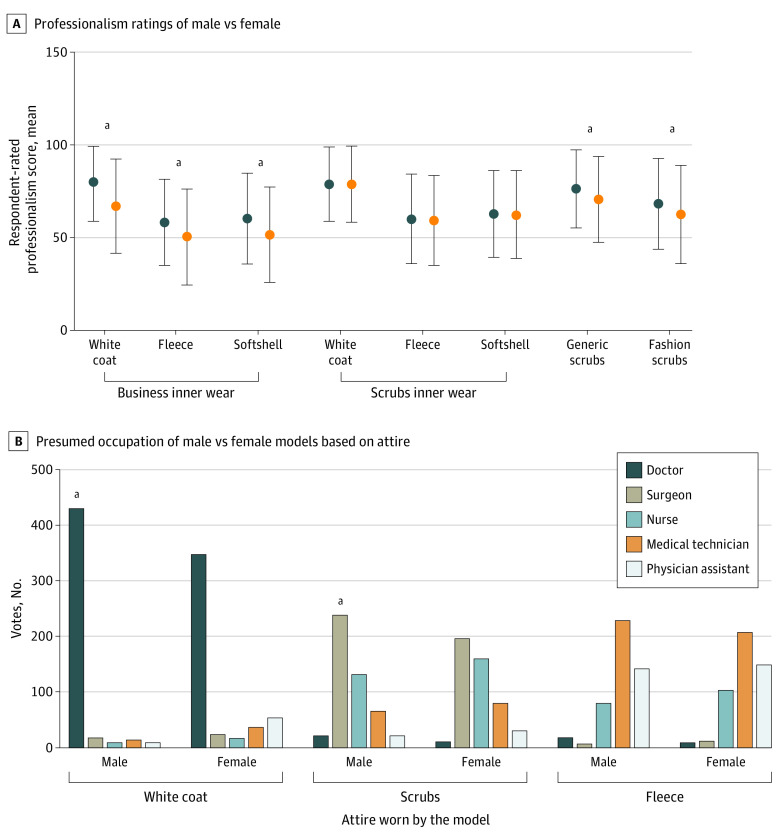
Professionalism Ratings and Presumed Occupations of Male Models vs Female Models by Attire A, Dots indicate mean values; whiskers, SDs. ^a^*P* < .001 for male models vs female models.

A male model wearing business inner wear with a white coat, fleece jacket, or softshell jacket was perceived as significantly more professional than a female model wearing the same attire (mean [SD] professionalism score: male, 65.8 [25.4]; female, 56.2 [20.2]; mean [SD] difference in professionalism score: white coat, 12.06 [1.10]; fleece, 7.89 [0.87]; softshell, 8.82 [0.93]; *P* < .001) (Figure 3A). A male model wearing hospital scrubs or fashion scrubs alone was also perceived as more professional than a female model in the same attire (mean [SD] difference in professionalism score: hospital scrubs, 5.68 [0.73]; fashion scrubs, 5.73 [0.89]; *P* < .001). The results of the multivariate analysis comparing professionalism scores for male and female models wearing the same attire across various rater characteristics are summarized in eAppendix 5 in the Supplement.

### Perceived Health Care Role by Attire and Gender of Photograph Model

We observed significant differences in the perceived health care roles for the male vs the female models (Figure 3B). Although the male and female models in white coats with business inner wear were most frequently perceived as physicians, the male model was more likely to be identified as a physician than was the female model (430 respondents [88.3%] for the male vs 349 [71.7%] for the female; *P* < .001) (eTable 2 in the Supplement). Compared with male models, female models in a white coat with business inner wear were more likely to be identified as a medical technician (39 [8.0%] vs 16 [3.3%]; *P* < .005) or a physician assistant (56 [11.5%] vs 11 [2.3%]; *P* < .001). Male and female models wearing hospital scrub attire alone were most frequently perceived as surgeons, but 241 respondents (49.5%) identified the male model wearing scrubs as a surgeon compared with 198 (40.7%) for the female model (*P* = .01); the female model in scrubs was more often perceived as a nurse (161 respondents [33.1%]) than was the male model (133 respondents [27.3%]) (*P* = .050).

## Discussion

To our knowledge, this is the first study to identify associations between gender, attire, and role identification in the health care environment. One finding of this study was the tendency of respondents to rate a model wearing either a gray fleece jacket or a black softshell jacket as less experienced and less professional compared with a model wearing a white coat. Lower experience ratings for casual physician attire may be explained by more limited exposure of the public to these garments owing to their novelty or by the association of these garments with younger wearers often still in medical training.^24^ Therefore, there have been few opportunities for the public to associate casual physician attire with valued physician characteristics. Populations with greater exposure to casual physician attire (younger respondent age and geographic location in the western US) were more accepting of it, potentially reflecting cultural shifts among certain populations.^3^ Although the results of the present survey showed a public preference for physicians in white coats compared with fleece or softshell jackets, these exposures and preference trends should be monitored longitudinally.

We expect casual physician attire to continue to increase in popularity for a multitude of reasons, including comfort, ease of transition between home and work, ease of laundering at home, and parallel trends in comfortable sportswear.^18,24^ Recently, the COVID-19 pandemic has provided additional impetus to forego the traditional white coat.^14^ However, the formal acknowledgment of the white coat’s role as a fomite^14^ may have led to the unintended further supplantation by casual physician attire, which in actuality may not be cleaner than white coats owing to a lack of regulations. As casual garments become more ubiquitous among all health care personnel, further research on sanitization standards will be critical, including garments’ roles as fomites and trends in hospital vs home wear.

For the second objective of this study, investigating gender biases, respondents rated a female model in business attire inner wear (with variable outerwear) as less professional compared with a male model (Figure 3), consistent with literature reporting that masculine features are perceived as more competent.^30^ An additional multivariate analysis revealed the association of key respondent characteristics, including gender, age, and geographic location, with gender-based professionalism ratings, thus highlighting the complexity and subjectivity of the perception of professionalism. One potential hypothesis is the lack-of-fit model^31^ in which individuals rate candidates based on preconceived archetypes of what a professional physician or surgeon should look like. In this model, diversity is penalized because the candidate is judged based on stereotype rather than merit. The stereotype used by a rater to determine whether a candidate is fit is formed and influenced by personal experiences, culture, and exposure to health care and can therefore be affected by experience and education.^31,32^

Gender-based physician stereotypes are also associated with role misidentification in health care, a phenomenon often reported anecdotally by female physicians having been mistaken as a nonphysician health care worker despite introductions and name tags.^25,26^ The findings of the present study suggest that female physicians are less likely than male physicians to be identified as physicians and more likely to be misidentified as nurses, medical technicians, or physician assistants. This misconception may be associated with prevailing stereotypes; although demographics in the US are shifting, most of the physician workforce is male (604 560 male vs 293 120 female),^33^ whereas the majority of the nursing workforce is female (3.2 million female vs 330 000 male).^34^ The public’s increased exposure to male physicians and female nurses contributes to the formation of gender-based professional stereotypes and associated biases. Such biases may lead to cumulative career disadvantages for female physicians^35^ or disadvantages that women encounter in their careers that result in more time spent on duties that do not provide additional career benefits.^36^ These disadvantages also include the time, focus, and resources used by female physicians to address gender biases and associated role misidentification.^25,26,37,38^ Most of the dialogue about gender bias in medicine has focused on interventions women can take to increase their identification as a physician, such as dressing more professionally and spending more time with patients to clarify their role or to build rapport.^39,40,41,42,43,44^ However, each of these interventions takes time and focus away from clinical work and may further be associated with the gender-based cumulative career disadvantages experienced by female physicians. Addressing gender bias is critical to promoting diversity and improving patient outcomes and should not be a responsibility solely undertaken by women; instead, it should be the responsibility of all health care workers to contribute to lasting change. Gender bias is especially prevalent in surgical disciplines,^45,46^ and institutional and organizational efforts are necessary to address obstacles and deterrents to recruitment and retainment of female surgeons.^47^

Physician attire is only a small aspect of the practice of medicine and does not embody the wearer’s qualifications, nor does it necessarily affect their performance, practice, and contributions. However, as physician attire evolves, the health care community should be attuned to the potential associations attire may have with the primary objective of the profession to provide excellent patient care. Integration of casual physician attire into daily practice should focus on building rapport with patients, reducing risks of nosocomial pathogen transmission, and communicating physicians’ role in patient care. All physicians, regardless of attire, should clarify their roles during introduction to patients and all team members. Physicians in casual physician attire should be conscious of the different impression they may give to patients compared with physicians in a white coat and mitigate this through other methods of building patient rapport. However, the introduction of new physician attire presents a disruptive opportunity to address persistent gender biases in medicine. With exposure and education, public perception of physicians can be broadened to reflect increasing diversity as the new status quo. This includes clear identification of professional roles during introductions, immediate correction of role misidentifications, and increased visibility (such as more diverse representation at all levels of training; spotlight features; representation on boards, as speakers, and in leadership positions; and presence on social media). This responsibility should not be undertaken only by the individuals that experience the biases, which may result in additional cumulative career disadvantages. The promotion of equality and diversity begins with recognition, characterization, and evidence-supported interventions and is a community operation.

### Limitations

This study has limitations. Owing to its cross-sectional design, the analysis was unable to follow trends in public perceptions of and gender biases in casual physician attire over time or to establish variable causation. An additional limitation was the lack of generalizability of findings owing to the survey population on MTurk. Most respondents were young (mean age, 36.2 years) and had a high educational level (49.9% had a bachelor’s degree). Despite this comparatively young respondent demographic, the respondents overall preferred traditional attire to casual attire. Furthermore, the respondent pool was not ideally balanced with respect to race/ethnicity (eg, 33 respondents [6.8%] were Black or African American) and socioeconomic status. However, the study’s purpose was to represent national demographics, and MTurk has been validated as a reliable and representative survey tool for health and medicine research^48^ with reliable internal consistency and test-retest reliability.^49,50,51^ Consequently, we believe MTurk was an acceptable platform for this pilot study to generate preliminary data and hypotheses to guide additional studies. The findings identified potential variables to guide more focused studies, such as patient surveys to identify specific preferences based on geographic location, setting of the medical practice, and gender of the physician. Furthermore, with the increasing use of telemedicine, initial impressions of and preferences for physician attire during virtual visits should be studied.

This study also used 1 male and 1 female model of the same race for the different attire combinations and did not include gender-nonconforming individuals or models from different racial groups. Although this method allowed control over appearance variabilities that could confound results, it potentially limited the generalizability of the findings. The survey asked to what extent respondents had prior personal interactions with female physicians or physicians of color; these personal interactions may be associated with respondents’ perceptions of physician professionalism in a diverse population of physicians and with the role of increased personal interactions in impression formation, but these associations were not evaluated in the present study. Nevertheless, the findings warrant future studies with more diverse groups of physicians to understand perceptions of physicians and potential interventions to reduce biases and support a culture of inclusivity and equal opportunity.

## Conclusions

In this survey study, survey respondents rated casual physician attire as being associated with less professionalism and experience compared with the traditional white coat. Female models in professional physician attire were rated as less professional and were less likely than their male counterparts to be identified as a physician. Multiple factors, such as age, gender, geographic location, and exposure to health care, were associated with perceptions of physician casual attire and gender biases. Future studies appear to be needed to further characterize biases and differences in public perceptions of physician attire and to identify interventions to address role confusion and cumulative career disadvantages for women in medicine.

## Notes

Supplement. **eAppendix 1.** Qualtrics survey distributed for this study **eAppendix 2.** Multivariate analysis of ranked health care provider values based on respondent characteristics **eAppendix 3.** Casual physician attire: public ratings of a physician’s professionalism, experience, and friendliness by physician attire and preference by profession **eAppendix 4.** Public preference of health care provider attire and variables impacting ratings **eAppendix 5.** Multivariate analysis of professionalism ratings for male and female model stratified by all raters, only male raters, and only female raters **eTable 1.** Respondent reported health care locations where the specific physician attire (white coat, scrubs, fleece jacket or vest, and softshell jacket) has been previously seen **eTable 2.** Respondents’ collated votes and percentages and *P* values between male vs female models

## References

[1] Shryock R. *The Development of Modern Medicine: An Interpretation of the Social and Scientific Factors Involved*. University of Pennsylvania Press; 1936.

[2] Hochberg MS. The doctor’s white coat: an historical perspective. *AMA Journal of Ethics*. April 2007. Accessed September 1, 2020. https://journalofethics.ama-assn.org/article/doctors-white-coat-historical-perspective/2007-04 10.1001/virtualmentor.2007.9.4.mhst1-0704 23217976

[3] Michael Petrilli C, Mack M, Janowitz Petrilli J, Hickner A, Saint S, Chopra V. Understanding the role of physician attire on patient perceptions: a systematic review of the literature—targeting attire to improve likelihood of rapport (TAILOR) investigators. BMJ Open. 2015;5(1):e006578. doi:10.1136/bmjopen-2014-006578 PMC4312788 25600254

[4] Au S, Khandwala F, Stelfox HT. Physician attire in the intensive care unit and patient family perceptions of physician professional characteristics. JAMA Intern Med. 2013;173(6):465-467. doi:10.1001/jamainternmed.2013.2732 23420343

[5] Mahida N. The white coat, microbiology service centralization, and combined infection training: what is happening to infection prevention and control? J Hosp Infect. 2015;91(4):289-291. doi:10.1016/j.jhin.2015.09.005 26520591

[6] Chung H, Lee H, Chang DS, . Doctor’s attire influences perceived empathy in the patient-doctor relationship. Patient Educ Couns. 2012;89(3):387-391. doi:10.1016/j.pec.2012.02.017 22445730

[7] Budny AM, Rogers LC, Mandracchia VJ, Lascher S. The physician’s attire and its influence on patient confidence. J Am Podiatr Med Assoc. 2006;96(2):132-138. doi:10.7547/0960132 16546951

[8] Wong D, Nye K, Hollis P. Microbial flora on doctors’ white coats. *BMJ*. 1991;303(6817):1602-1604. doi:10.1136/bmj.303.6817.1602 PMC1676235 1773186

[9] Selwyn S. Mackie and McCartney: Practical Medical Microbiology—book review. J Hosp Infect. 1990;15(2):201. doi:10.1016/0195-6701(90)90135-b

[10] Varghese D, Patel H. Hand washing: stethoscopes and white coats are the sources of nosocomial infections. *BMJ*. 1999;319(7208):519. 10507865

[11] Neely AN. A survey of gram-negative bacteria survival on hospital fabrics and plastics. J Burn Care Rehabil. 2000;21(6):523-527. doi:10.1097/00004630-200021060-00009 11194806

[12] Treakle AM, Thom KA, Furuno JP, Strauss SM, Harris AD, Perencevich EN. Bacterial contamination of health care workers’ white coats. Am J Infect Control. 2009;37(2):101-105. doi:10.1016/j.ajic.2008.03.009 18834751 PMC2892863

[13] Haun N, Hooper-Lane C, Safdar N. Healthcare personnel attire and devices as fomites: a systematic review. Infect Control Hosp Epidemiol. 2016;37(11):1367-1373. doi:10.1017/ice.2016.192 27609491

[14] Tse G, Withey JM, Yeo CA. Bare below the elbows: was the target the white coat? *J Hosp Infec*. 2015,91(4):299-301. doi:10.1016/j.jhin.2015.08.003 10.1016/j.jhin.2015.08.003 26364208

[15] Major K, Hayase Y, Balderrama D, Lefor AT. Attitudes regarding surgeons’ attire. Am J Surg. 2005;190(1):103-106. doi:10.1016/j.amjsurg.2005.04.003 15972180

[16] Banu A, Anand M, Nagi N. White coats as a vehicle for bacterial dissemination. J Clin Diagn Res. 2012;6(8):1381-1384. doi:10.7860/JCDR/2012/4286.2364 23205352 PMC3471503

[17] Limeri D. Medicine Is a Team Sport: How to Become a Team Player. CreateSpace Independent Publishing Platform; 2012.

[18] Prentiss Ott K. This is what I’m wearing instead of a white coat. *KevinMD*. February 16, 2016. Accessed September 1, 2020. https://www.kevinmd.com/blog/2016/02/im-wearing-instead-white-coat.html

[19] Rehman SU, Nietert PJ, Cope DW, Kilpatrick AO. What to wear today? effect of doctor’s attire on the trust and confidence of patients. Am J Med. 2005;118(11):1279-1286. doi:10.1016/j.amjmed.2005.04.026 16271913

[20] Kurihara H, Maeno T, Maeno T. Importance of physicians’ attire: factors influencing the impression it makes on patients, a cross-sectional study. Asia Pac Fam Med. 2014;13(1):2. doi:10.1186/1447-056X-13-2 24397871 PMC3890493

[21] Fischer RL, Hansen CE, Hunter RL, Veloski JJ. Does physician attire influence patient satisfaction in an outpatient obstetrics and gynecology setting? Am J Obstet Gynecol. 2007;196(2):186.e1-186.e5. doi:10.1016/j.ajog.2006.09.043 17306675

[22] Mountain High Outfitters. Patagonia women’s better sweater 1/4 zip fleece pullover. Accessed September 20, 2020. https://mountainhighoutfitters.com/womens-better-sweater-14-zip-fleece-25616.html

[23] Decathlon. Women’s backpacking softshell jacket Trek 100. Accessed September 20, 2020. https://www.decathlon.com/products/womens-hiking-softshell-jacket-windwarm-100?variant=19061258584126&currency=USD&utm_medium=product_sync&utm_source=google&utm_content=sag_organic&utm_campaign=sag_organic&gclid=CjwKCAjw-5v7BRAmEiwAJ3DpuGmpSigzThdLGkuWQ4a

[24] Ostfeld-Johns S. New interns: get ready to be fleeced. *KevinMD*. August 11, 2018. Accessed September 1, 2020. https://www.kevinmd.com/blog/2018/08/new-interns-get-ready-to-be-fleeced.html

[25] Tran-Harding K. The physician gender bias—what every female has faced. April 2, 2018. Updated February 16, 2019. Accessed June 12, 2021. https://www.studentdoctor.net/2018/04/03/the-physician-gender-bias-what-every-female-doctor-has-faced/

[26] Tran-Harding K. Oh, are you a nurse? the physician gender bias. *KevinMD*. April 26, 2018. Accessed September 1, 2020. https://www.kevinmd.com/blog/2018/04/oh-are-you-a-nurse-the-physician-gender-bias.html

[27] Jennings JD, Ciaravino SG, Ramsey FV, Haydel C. Physicians’ attire influences patients’ perceptions in the urban outpatient orthopaedic surgery setting. Clin Orthop Relat Res. 2016;474(9):1908-1918. doi:10.1007/s11999-016-4855-7 27116208 PMC4965372

[28] Jennings JD, Pinninti A, Kakalecik J, Ramsey FV, Haydel C. Orthopaedic physician attire influences patient perceptions in an urban inpatient setting. Clin Orthop Relat Res. 2019;477(9):2048-2058. doi:10.1097/CORR.0000000000000822 31294719 PMC7000081

[29] Fox JD, Prado G, Baquerizo Nole KL, . Patient preference in dermatologist attire in the medical, surgical, and wound care settings. JAMA Dermatol. 2016;152(8):913-919. doi:10.1001/jamadermatol.2016.1186 27248428

[30] Oh D, Buck EA, Todorov A. Revealing hidden gender biases in competence impressions of faces. Psychol Sci. 2019;30(1):65-79. doi:10.1177/0956797618813092 30526301

[31] Heilman ME, Caleo S. Combatting gender discrimination: a lack of fit framework. *Gr Process Intergr Relations*. Published online 2018;21(5):725-744. doi:10.1177/1368430218761587

[32] Heilman ME. Gender stereotypes and workplace bias. Res Organ Behav. 2012;32:113-135. doi:10.1016/j.riob.2012.11.003

[33] Association of American Medical Colleges. 2019 State physician workforce *data report*. Association of American Medical Colleges; 2019. Accessed June 18, 2021. https://www.aamc.org/data-reports/workforce/report/state-physician-workforce-data-report

[34] Landivar LC. Men in nursing occupations. US Census Bureau. February 25, 2013. Accessed September 1, 2020. https://www.census.gov/newsroom/blogs/random-samplings/2013/02/men-in-nursing-occupations.html

[35] Colletti LM, Mulholland MW, Sonnad SS. Perceived obstacles to career success for women in academic surgery. Arch Surg. 2000;135(8):972-977. doi:10.1001/archsurg.135.8.972 10922261

[36] Bickel J. Maximizing professional development of women in academic medicine. *The Scientist*. May 1997. Accessed September 1, 2020. https://www.the-scientist.com/commentary/maximizing-professional-development-of-women-in-academic-medicine-57487

[37] Lemay MS. Thanks for the compliment, but I’m not a nurse. *Slate*. September 20, 2013. Accessed June 18, 2021. https://slate.com/human-interest/2013/09/i-m-not-a-nurse-i-m-a-female-doctor-but-thanks-for-the-compliment.html

[38] Fogel J. Unpacking the “insult” of being called a nurse as a female physician. *in-Training*. March 2, 2020. Accessed September 1, 2020. https://in-training.org/unpacking-the-insult-of-being-called-a-nurse-as-a-female-physician-19041

[39] de Costa J, Chen-Xu J, Bentounsi Z, Vervoort D. Women in surgery: challenges and opportunities. *IJS Global Health*. 2018;1(1):e02. doi:10.1097/GH9.0000000000000002

[40] Healy NA, Cantillon P, Malone C, Kerin MJ. Role models and mentors in surgery. Am J Surg. 2012;204(2):256-261. doi:10.1016/j.amjsurg.2011.09.031 22621833

[41] Fried LP, Francomano CA, MacDonald SM, . Career development for women in academic medicine: multiple interventions in a department of medicine. JAMA. 1996;276(11):898-905. doi:10.1001/jama.1996.03540110052031 8782639

[42] Hoobler J, Wayne S, Lemmon G. Bosses’ perceptions of family-work conflict and women’s promotability: glass ceiling effects. Acad Manag J. 2009;52(5):939-957. doi:10.5465/amj.2009.44633700

[43] Incorvaia AN, Ringley CD, Boysen DA. Factors influencing surgical career decisions. Curr Surg. 2005;62(4):429-435. doi:10.1016/j.cursur.2005.02.002 15964470

[44] Lee SK, Chang J, Kadakia N, . Trends in participation and authorship of women at the Pacific Coast Surgical Association Meeting, 2008-2018. *JAMA Surg*. 2020;155(9):891-893. 10.1001/jamasurg.2020.2182 PMC7364380 32667661

[45] Gargiulo DA, Hyman NH, Hebert JC. Women in surgery: do we really understand the deterrents? Arch Surg. 2006;141(4):405-407. doi:10.1001/archsurg.141.4.405 16618901

[46] Wirtzfeld DA. The history of women in surgery. Can J Surg. 2009;52(4):317-320. doi:10.1016/S0008-428X(09)50102-6 19680519 PMC2724816

[47] Morgan AU, Chaiyachati KH, Weissman GE, Liao JM. Eliminating gender-based bias in academic medicine: more than naming the “elephant in the room.” J Gen Intern Med. 2018;33(6):966-968. doi:10.1007/s11606-018-4411-0 29564608 PMC5975172

[48] Mortensen K, Hughes TL. Comparing Amazon’s Mechanical Turk platform to conventional data collection methods in the health and medical research literature. J Gen Intern Med. 2018;33(4):533-538. doi:10.1007/s11606-017-4246-0 29302882 PMC5880761

[49] Arditte KA, Çek D, Shaw AM, Timpano KR. The importance of assessing clinical phenomena in Mechanical Turk research. Psychol Assess. 2016;28(6):684-691. doi:10.1037/pas0000217 26302105

[50] Chandler J, Shapiro D. Conducting clinical research using crowdsourced convenience samples. Annu Rev Clin Psychol. 2016;12:53-81. doi:10.1146/annurev-clinpsy-021815-093623 26772208

[51] Buhrmester M, Kwang T, Gosling SD. Amazon’s Mechanical Turk: a new source of inexpensive, yet high-quality, data? Perspect Psychol Sci. 2011;6(1):3-5. doi:10.1177/1745691610393980 26162106

