# Supplementary material for: Public Perceptions of Physician Attire and Professionalism in the US
Source: JAMA Netw Open. 2021 Jul 30;4(7):e2117779. doi: 10.1001/jamanetworkopen.2021.17779 (PMC8325071; doi:10.1001/jamanetworkopen.2021.17779)
Supplement: Supplement. — eAppendix 1. Qualtrics survey distributed for this study eAppendix 2. Multivariate analysis of ranked health care provider values based on respondent characteristics eAppendix 3. Casual physician attire: public ratings of a physician’s professionalism, experience, and friendliness by physician attire and preference by profession eAppendix 4. Public preference of health care provider attire and variables impacting ratings eAppendix 5. Multivariate analysis of professionalism ratings for male and female model stratified by all raters, only male raters, and only female raters eTable 1. Respondent reported health care locations where the specific physician attire (white coat, scrubs, fleece jacket or vest, and softshell jacket) has been previously seen eTable 2. Respondents’ collated votes and percentages and P values between male vs female models [file jamanetwopen-e2117779-s001.pdf]

Supplementary Online Content

Xun H, Chen J, Sun AH, Jenny HE, Liang F, Steinberg JP. Public perceptions of physician attire and professionalism in the US. *JAMA Netw Open*. 2021;4(7):e2117779. doi:10.1001/jamanetworkopen.2021.17779

eAppendix 1. Qualtrics survey distributed for this study
eAppendix 2. Multivariate analysis of ranked health care provider values based on respondent characteristics
eAppendix 3. Casual physician attire: public ratings of a physician's professionalism, experience, and friendliness by physician attire and preference by profession
eAppendix 4. Public preference of health care provider attire and variables impacting ratings
eAppendix 5. Multivariate analysis of professionalism ratings for male and female model stratified by all raters, only male raters, and only female raters
eTable 1. Respondent reported health care locations where the specific physician attire (white coat, scrubs, fleece jacket or vest, and softshell jacket) has been previously seen
eTable 2. Respondents' collated votes and percentages and *P* values between male vs female models

This supplementary material has been provided by the authors to give readers additional information about their work.

© 2021 Xun H et al. *JAMA Network Open*.

eAppendix 1. Qualtrics Survey distributed for this study. This Qualtrics™ Survey was crowdsourced using Amazon Mechanical Turks. Respondents were notified that continuing with the survey would serve as informed consent for study participation. Surveys were terminated if the respondent did not correctly pass the CAPTCHA, or excluded from analysis if one of the two attention check questions (one multiple choice, one sliding bar) were answered incorrectly. Respondents were compensated $0.25 per survey.

Professional attire for Healthcare Personnel

Start of Block: Default Question Block

Q1 In the following survey, you will answer several questions regarding your opinions on professional attire. There is no right or wrong answer. This survey should take 10 minutes to complete. Please DO NOT take this survey if you are under the age of 18 or if English is not your primary language. All responses will be recorded confidentially and stored securely. Your completion of the survey will serve as your consent to be in this research study. Upon analyzing the data, all identifying information will be removed and the data will be analyzed in aggregate to further protect the privacy of your responses. Please contact hxun1@jhmi.edu if you have any questions or concerns. We sincerely appreciate your participation!

Study principal investigator: Dr. Jordan Steinberg
IRB00243781

End of Block: Default Question Block

Start of Block: Captcha

Q133

End of Block: Captcha

Start of Block: Exposure to medicine

Q31 How many times in the past year have you visited the following? Use your best estimate.

	10+ times

	0	1	2	3	4	5	6	7	8	9	10

© 2021 Xun H et al. *JAMA Network Open*.

Family doctor or primary care physician ()	
Internal medicine doctor and specialists (cardiologists, nephrologists, pulmonologists, et cetera) ()	
Emergency room or urgent care ()	
Dermatology or cosmetic plastic surgery ()	
Physical therapy or occupational therapy ()	
Surgeon ()	

Q32 Have you ever been admitted to the hospital in any capacity as a patient? This means you spent the night at the hospital as a patient.

- Yes (1)
- No (3)

Display This Question:

If Have you ever been admitted to the hospital in any capacity as a patient? This means you spent th... = Yes

© 2021 Xun H et al. *JAMA Network Open*.

Q33 Select the statement that best describes the number of days you are admitted into the hospital a year. This means you spent the night at the hospital as a patient.

- Less than once a year (1)
- 1-2 times a year (2)
- 3-10 times a year (3)
- 10-20 times a year (4)
- More than 20 times a year (5)

Q36 Do you work in healthcare settings?

- Yes (1)
- No (2)
- Not sure (3)

Display This Question:

If Do you work in healthcare settings? = Yes

Q37 What is your role in healthcare?

- Provider (1)
- Administrator (2)
- Support Staff (3)

© 2021 Xun H et al. *JAMA Network Open*.

Q38 Does your significant other or a family member work in healthcare settings?

◯ Yes (1)

◯ No (2)

◯ Not sure (3)

Display This Question:

If Does your significant other or a family member work in healthcare settings? = Yes

Q40 What is their role in healthcare?

◯ Provider (1)

◯ Administrator (2)

◯ Support (3)

Q47 Have you ever had a surgery before for which you were put to sleep?

◯ Yes (1)

◯ No (2)

Q48 Select the option "3" for this question.

◯ 1 (1)

◯ 2 (2)

◯ 3 (3)

End of Block: Exposure to medicine

© 2021 Xun H et al. *JAMA Network Open*.

Start of Block: Professional Attire

Q77 When you visit healthcare providers, how often do you see them wearing white coats?

- Always (1)
- Most of the time (2)
- Sometimes (3)
- Rarely (4)
- Never (5)

Q45 When you visit healthcare providers, how often do you see them wearing scrubs?

- Always (1)
- Most of the time (2)
- Sometimes (3)
- Rarely (4)
- Never (5)

Q49 When you visit healthcare providers, how often do you see them wearing fleece blended sweaters or vests?

© 2021 Xun H et al. *JAMA Network Open*.

○ Always (1)

○ Most of the time (2)

○ Sometimes (3)

○ Rarely (4)

○ Never (5)

Q51 When you visit healthcare providers, how often do you see them wearing smooth softshell jackets (black jacket pictured below)?

○ Always (1)

○ Most of the time (2)

○ Sometimes (3)

○ Rarely (4)

○ Never (5)

Q73 Please select all environments in which you have seen a healthcare provider wear a white coat.

© 2021 Xun H et al. *JAMA Network Open*.

- [ ] Outpatient clinic (1)
- [ ] Inpatient hospital wards (2)
- [ ] Emergency room/urgent care (3)
- [ ] Surgical areas: outpatient surgical area, surgicenter, perioperative areas (4)

Q74
Please select all environments in which you have seen a healthcare provider wear scrubs.

- [ ] Outpatient clinic (1)
- [ ] Inpatient hospital wards (2)
- [ ] Emergency room/urgent care (3)
- [ ] Surgical areas: outpatient surgical area, surgicenter, perioperative areas (4)

Q75 Please select all environments in which you have seen a healthcare provider wear a fleece blended sweater or vest.

© 2021 Xun H et al. *JAMA Network Open*.

- ☐ Outpatient clinic (1)
- ☐ Inpatient hospital wards (2)
- ☐ Emergency room/urgent care (3)
- ☐ Surgical areas: outpatient surgical area, surgicenter, perioperative areas (4)

Q76 Please select all environments in which you have seen a healthcare provider wear a softshell jacket.

- ☐ Outpatient clinic (1)
- ☐ Inpatient hospital wards (2)
- ☐ Emergency room/urgent care (3)
- ☐ Surgical areas: outpatient surgical area, surgicenter, perioperative areas (4)

© 2021 Xun H et al. *JAMA Network Open*.

Q42 Order the following healthcare providers from most EXPERIENCED (1) to least EXPERIENCED (6).
______ (1)
______ (2)
______ (3)
______ (4)
______ (5)
______ (6)

Q81 Order the following healthcare providers from most PROFESSIONAL (1) to least PROFESSIONAL (6) .
______ (1)
______ (2)
______ (3)
______ (4)
______ (5)
______ (6)

Q82 Order the following healthcare providers from most FRIENDLY (1) to least FRIENDLY (6) .
______ (1)
______ (2)
______ (3)
______ (4)
______ (5)
______ (6)

Q58 When thinking about your healthcare providers, rank the following from most important (1) to least important (3) .
______ Friendly (1)
______ Professional (2)
______ Experienced (3)

End of Block: Professional Attire

© 2021 Xun H et al. *JAMA Network Open*.

Start of Block: PROFESSION AND SPECIALTY DEPENDENT

Q56 A nurse walks into your patient room. What would you prefer to see him or her wear?

______ (1)
______ (2)
______ (3)
______ (4)
______ (5)
______ (6)
______ Image:Dsc00600 small (8)
______ Image:Dsc00600 withname small (9)

Q84 A technician walks into your patient room to take your blood pressure. What would you prefer to see him or her wear?

______ (1)
______ (2)
______ (3)
______ (4)
______ (5)
______ (6)
______ Image:Dsc00600 small (7)
______ Image:Dsc00600 withname small (8)

© 2021 Xun H et al. *JAMA Network Open*.

Q85 A phlebotomist walks into your patient room to draw your blood. What would you prefer to see him or her wear?

______ (1)
______ (2)
______ (3)
______ (4)
______ (5)
______ (6)
______ Image:Dsc00600 small (7)
______ Image:Dsc00600 withname small (8)

Q86 A family doctor walks into your patient room. What would you prefer to see him or her wear?

______ (1)
______ (2)
______ (3)
______ (4)
______ (5)
______ (6)
______ Image:Dsc00600 small (7)
______ Image:Dsc00600 withname small (8)

Q87 A surgeon walks into your patient room. What would you prefer to see him or her wear?

______ (1)
______ (2)
______ (3)
______ (4)
______ (5)
______ (6)
______ Image:Dsc00600 small (7)
______ Image:Dsc00600 withname small (8)

© 2021 Xun H et al. *JAMA Network Open*.

Q88 A dermatologist walks into your patient room. What would you prefer to see him or her wear?

______ (1)

______ (2)

______ (3)

______ (4)

______ (5)

______ (6)

______ Image:Dsc00600 small (7)

______ Image:Dsc00600 withname small (8)

End of Block: PROFESSION AND SPECIALTY DEPENDENT

Start of Block: GENDER

Q93

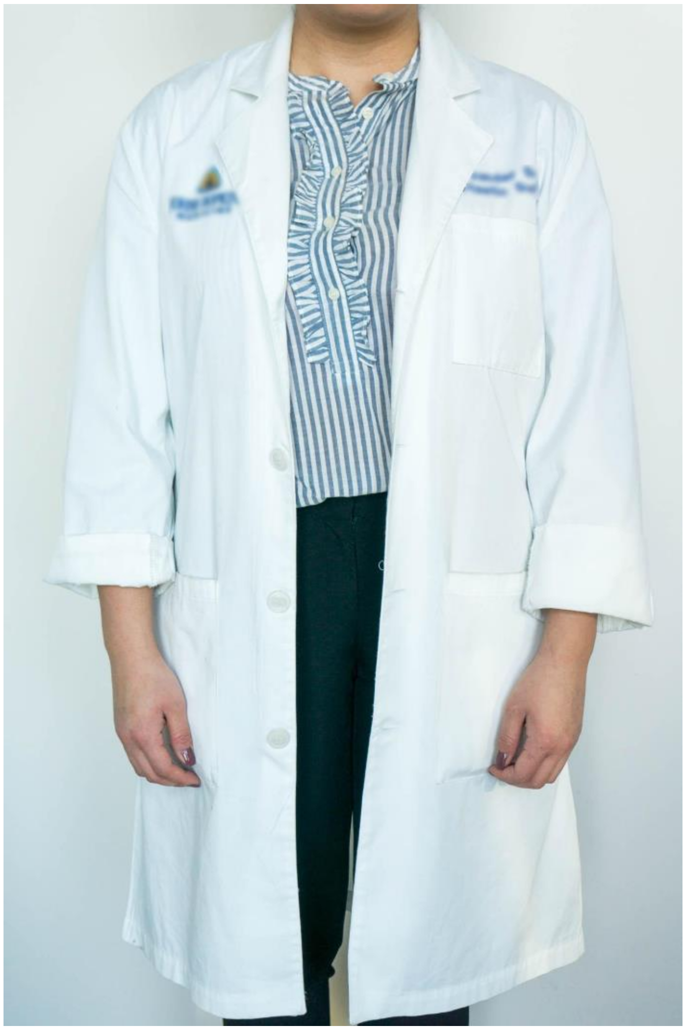

© 2021 Xun H et al. *JAMA Network Open*.

Q94 Please rate the professionalism of the subject above from 0 (least professional) to 100 (most professional).

	0 10 20 30 40 50 60 70 80 90 100
Professionalism ()	

Q95

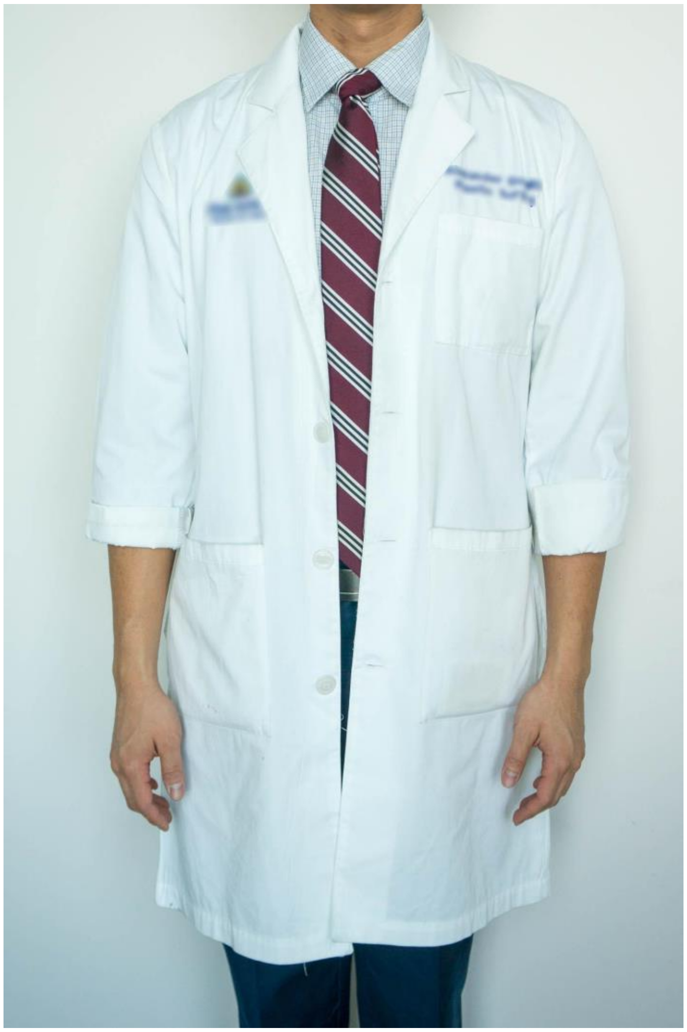

© 2021 Xun H et al. *JAMA Network Open*.

Q96 Please rate the professionalism of the subject above from 0 (least professional) to 100 (most professional).

	0 10 20 30 40 50 60 70 80 90 100
Professionalism ()	

Q97

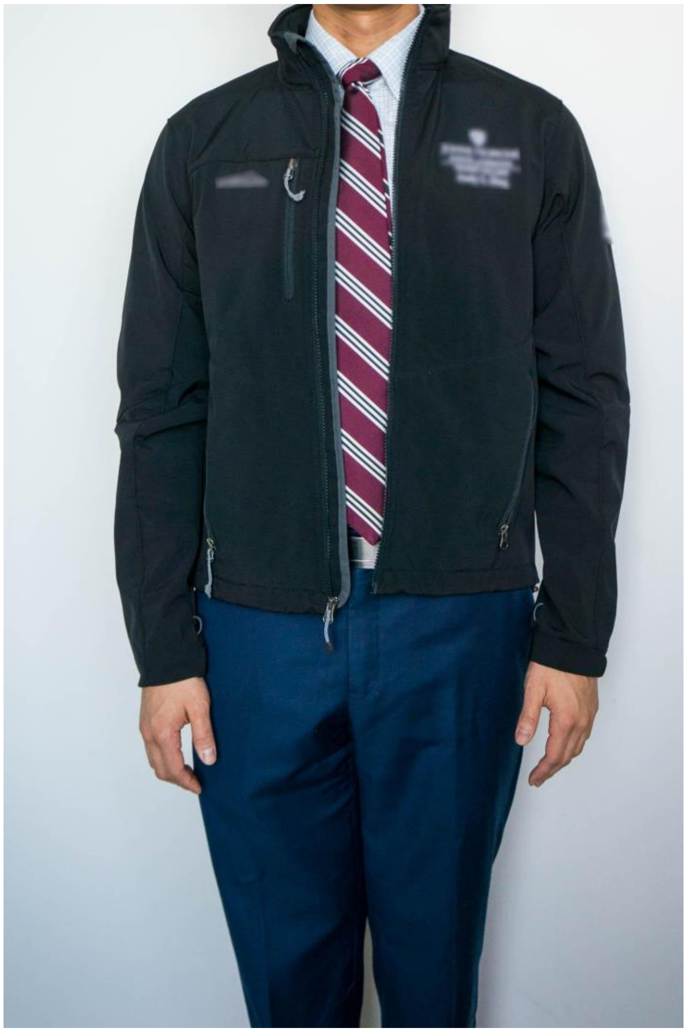

© 2021 Xun H et al. *JAMA Network Open*.

Q98 Please rate the professionalism of the subject above from 0 (least professional) to 100 (most professional).

	0	10	20	30	40	50	60	70	80	90	100
Professionalism ()											

Q99

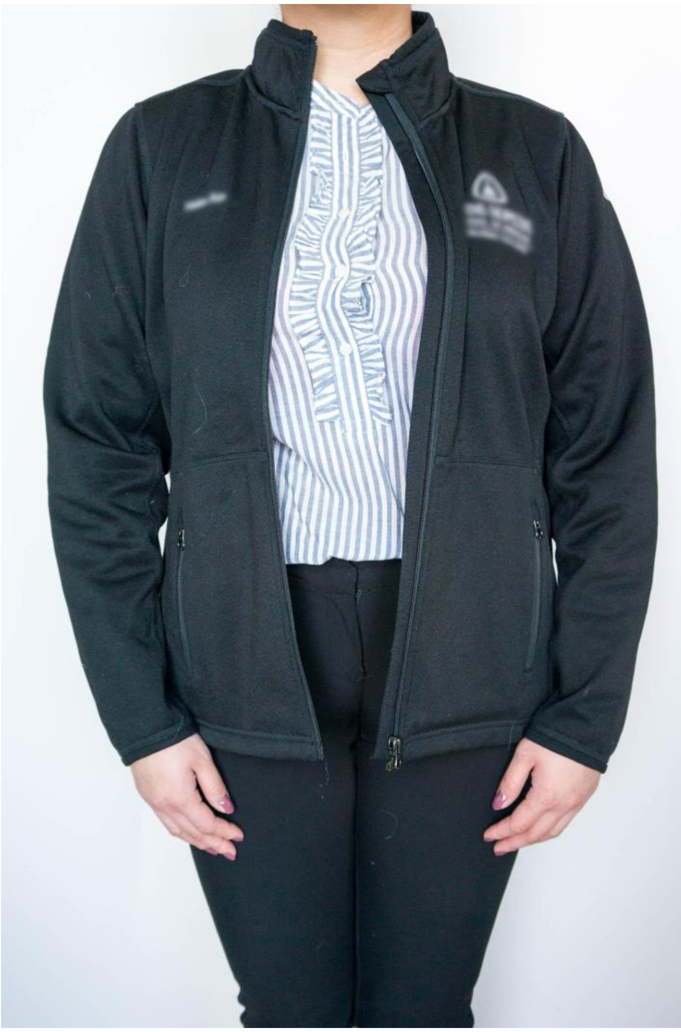

© 2021 Xun H et al. *JAMA Network Open*.

Q100 Please rate the professionalism of the subject above from 0 (least professional) to 100 (most professional).

	0 10 20 30 40 50 60 70 80 90 100
Professionalism ()	

Q118

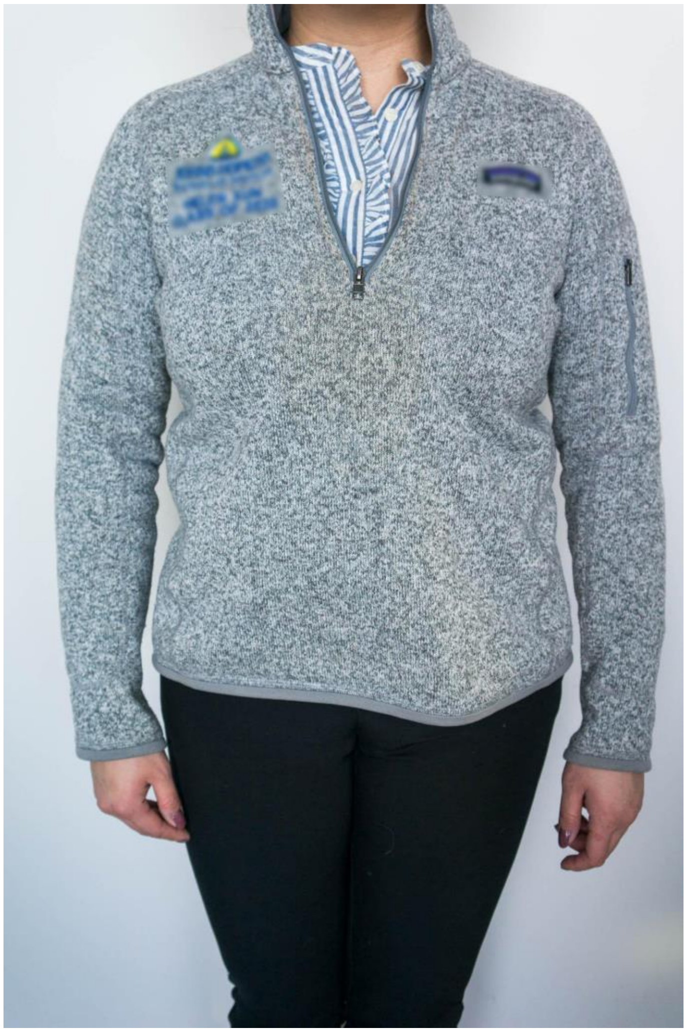

© 2021 Xun H et al. *JAMA Network Open*.

Q119 Please rate the professionalism of the subject above from 0 (least professional) to 100 (most professional).

	0	10	20	30	40	50	60	70	80	90	100
Professionalism ()											

Q120

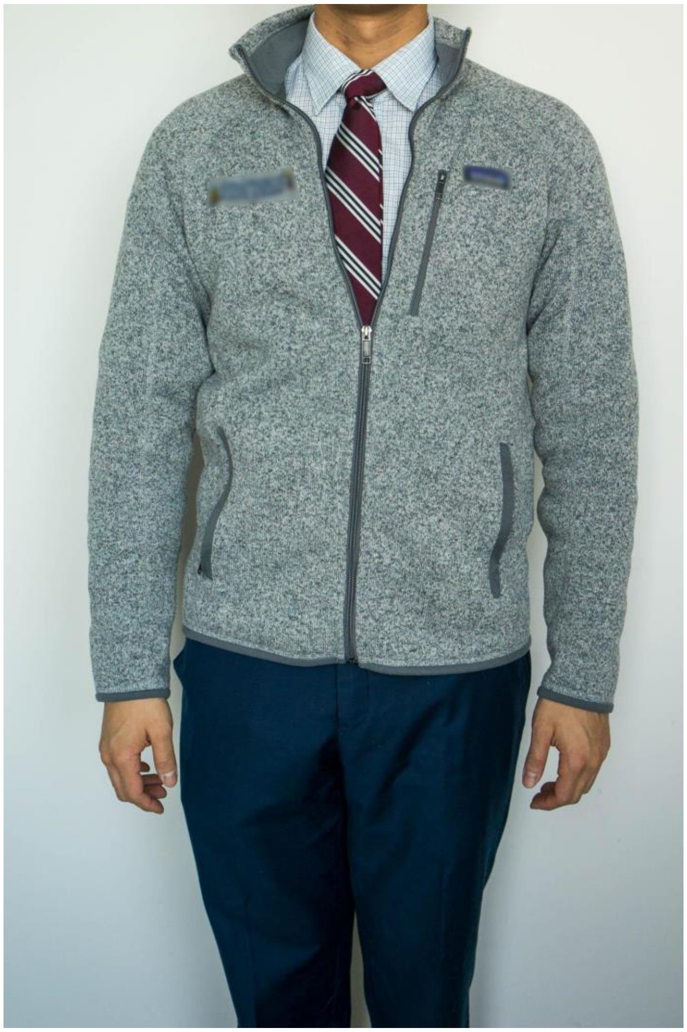

© 2021 Xun H et al. *JAMA Network Open*.

Q121 Please rate the professionalism of the subject above from 0 (least professional) to 100 (most professional).

	0 10 20 30 40 50 60 70 80 90 100
Professionalism ()	

Q101

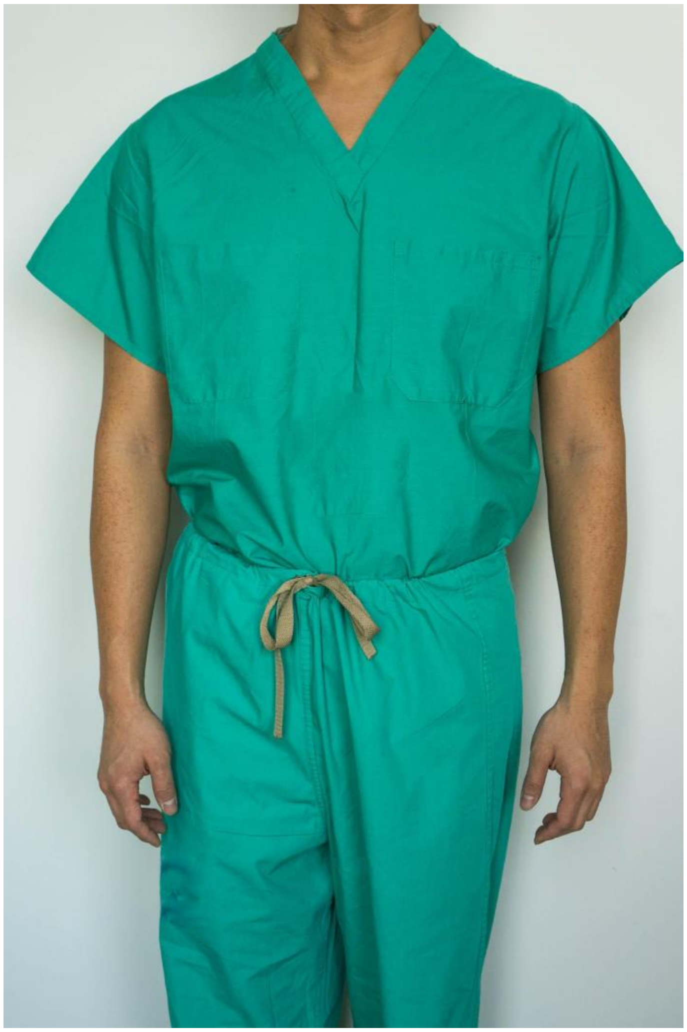

© 2021 Xun H et al. *JAMA Network Open*.

Q102 Please rate the professionalism of the subject above from 0 (least professional) to 100 (most professional).

	0	10	20	30	40	50	60	70	80	90	100
Professionalism ()											

Q103

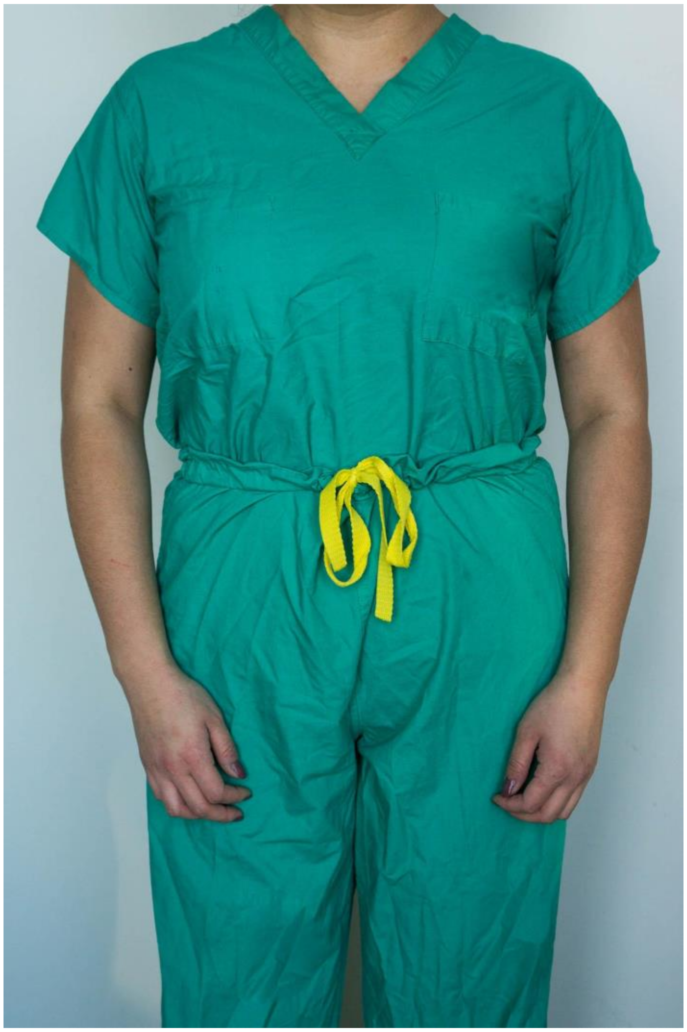

© 2021 Xun H et al. *JAMA Network Open*.

Q104 Please rate the professionalism of the subject above from 0 (least professional) to 100 (most professional).

	0 10 20 30 40 50 60 70 80 90 100
Professionalism ()	

Q105

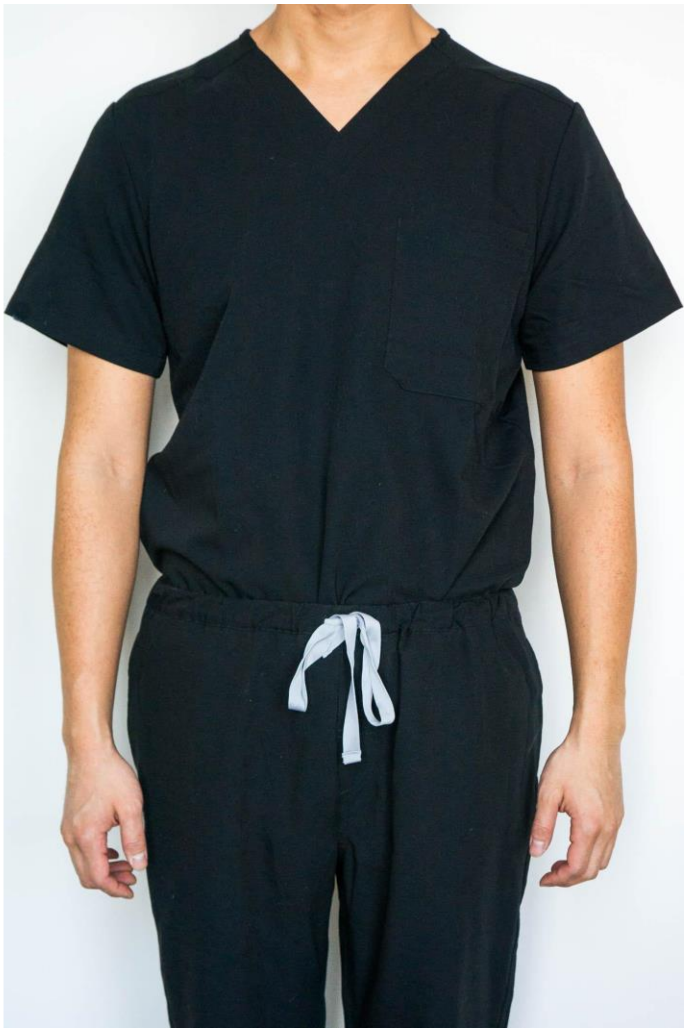

© 2021 Xun H et al. *JAMA Network Open*.

Q106 Please rate the professionalism of the subject above from 0 (least professional) to 100 (most professional).

	0 10 20 30 40 50 60 70 80 90 100
Professionalism ()	

Q109

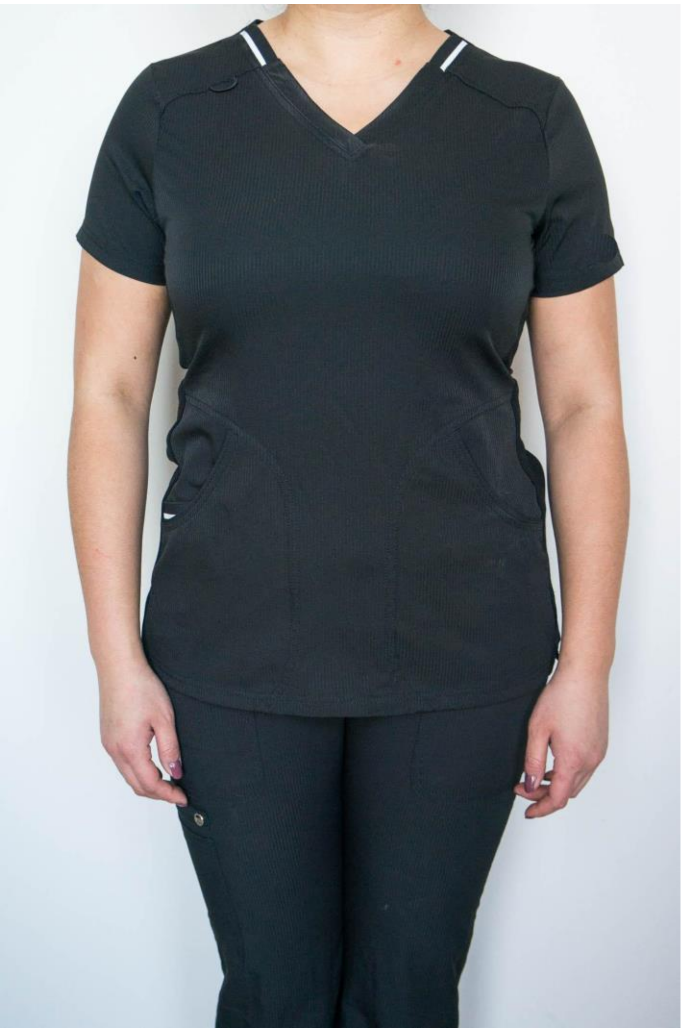

© 2021 Xun H et al. *JAMA Network Open*.

Q108 Please rate the professionalism of the subject above from 0 (least professional) to 100 (most professional).

	0 10 20 30 40 50 60 70 80 90 100
Professionalism ()	

Q110

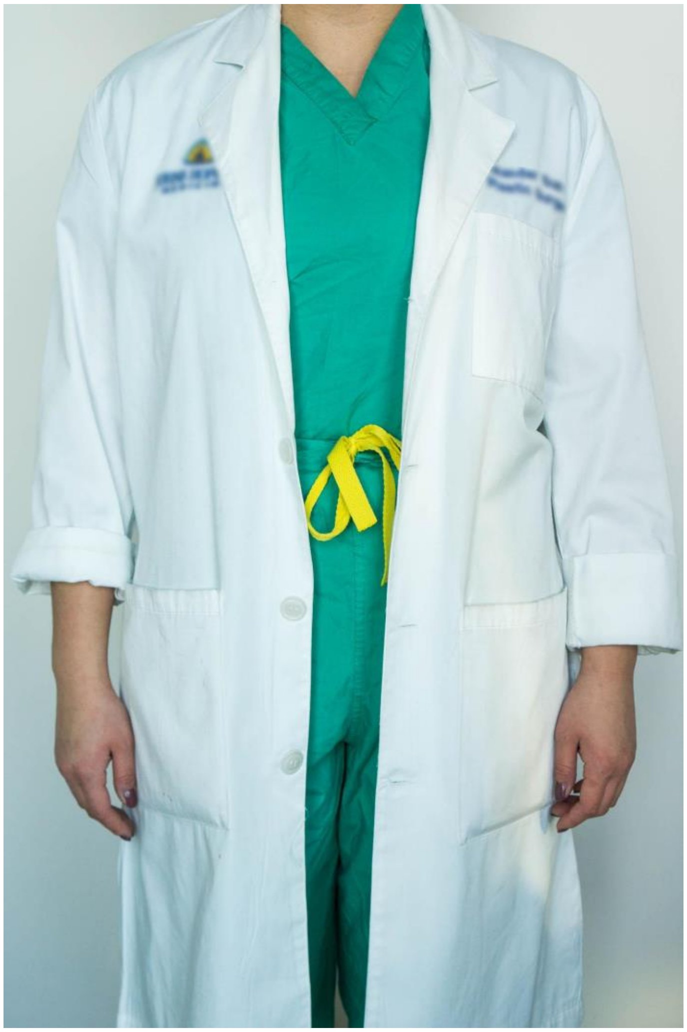

© 2021 Xun H et al. *JAMA Network Open*.

Q111 Please rate the professionalism of the subject above from 0 (least professional) to 100 (most professional).

	0	10	20	30	40	50	60	70	80	90	100
Professionalism ()											

Q112

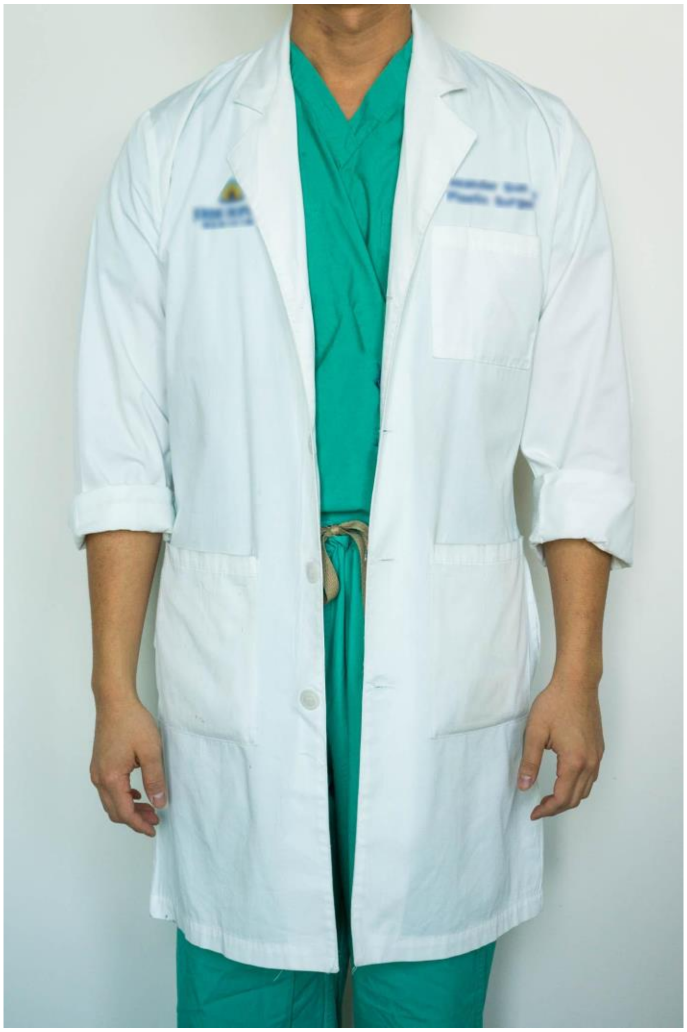

© 2021 Xun H et al. *JAMA Network Open*.

Q113 Please rate the professionalism of the subject above from 0 (least professional) to 100 (most professional).

0 10 20 30 40 50 60 70 80 90 100

Professionalism ()	

Q114

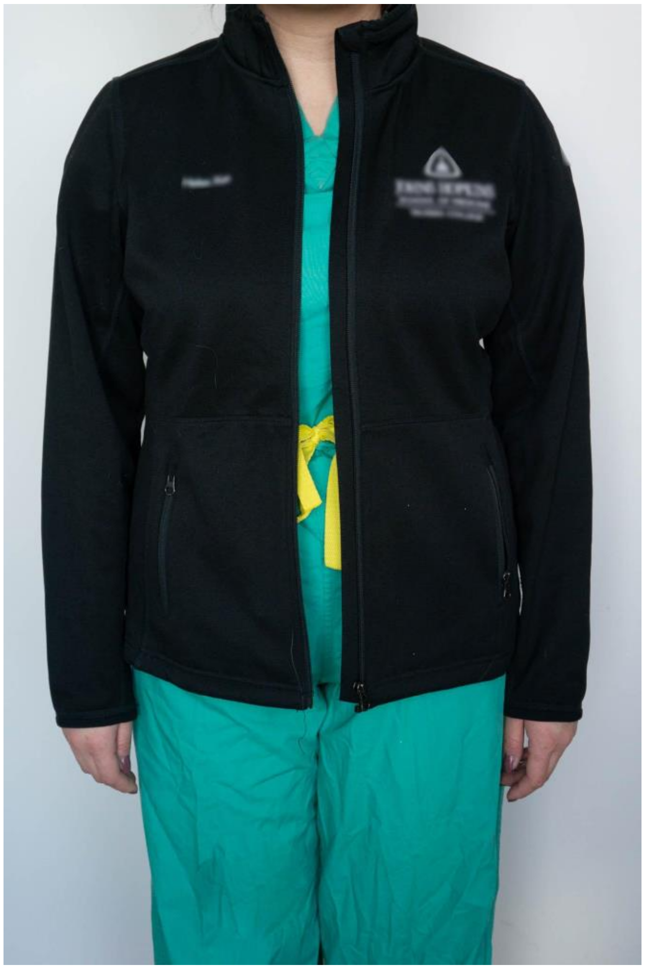

Q115 Please rate the professionalism of the subject above from 0 (least professional) to 100 (most professional).

0 10 20 30 40 50 60 70 80 90 100

© 2021 Xun H et al. *JAMA Network Open*.

Professionalism ()	

Q116

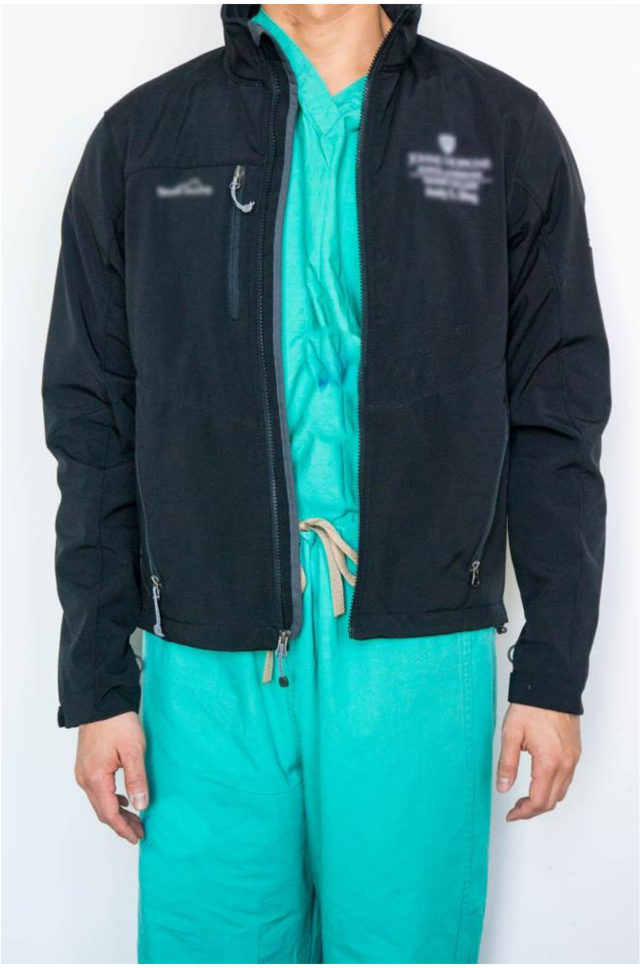

Q117 Please rate the professionalism of the subject above from 0 (least professional) to 100 (most professional).

	0	10	20	30	40	50	60	70	80	90	100
Professionalism ()											

© 2021 Xun H et al. *JAMA Network Open*.

Q122

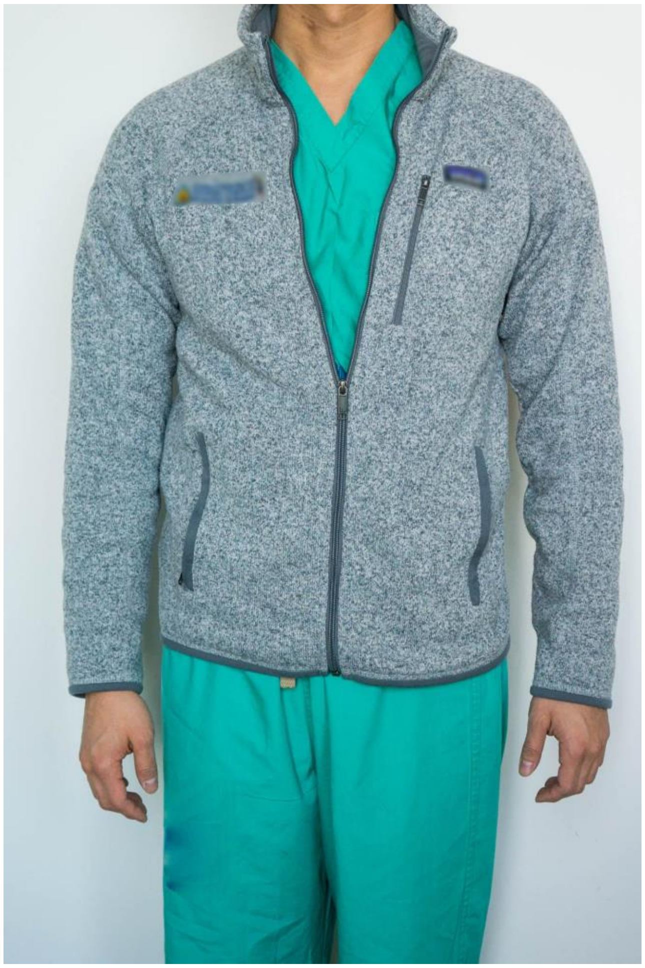

Q123 Please rate the professionalism of the subject above from 0 (least professional) to 100 (most professional).

	0	10	20	30	40	50	60	70	80	90	100
Professionalism ()											

© 2021 Xun H et al. *JAMA Network Open*.

Q124

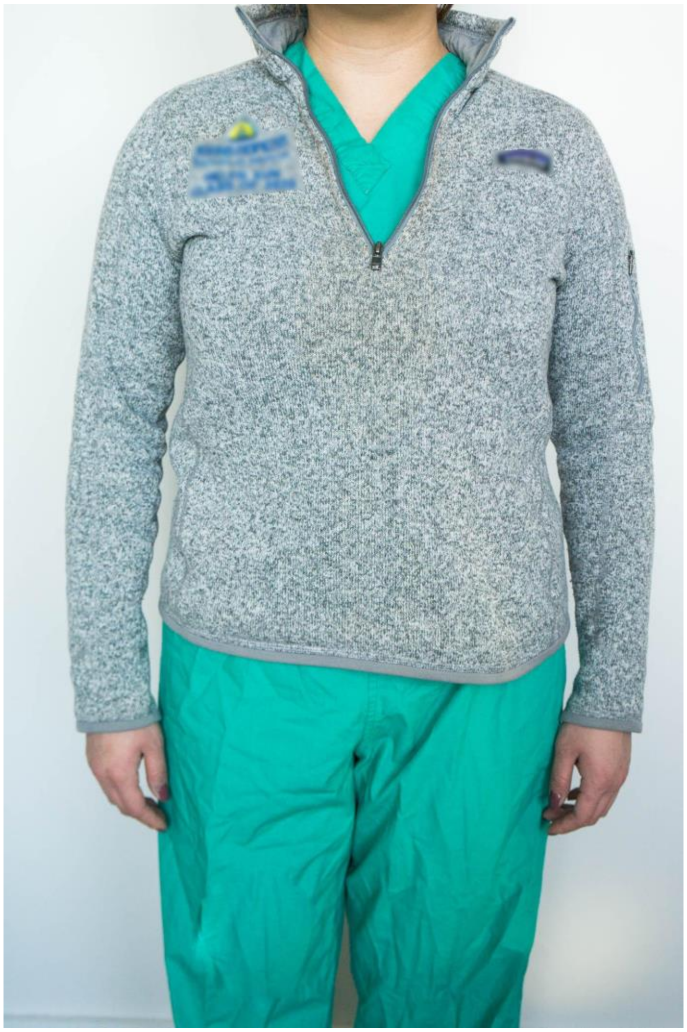

Q125 Please rate the professionalism of the subject above from 0 (least professional) to 100 (most professional).

	0 10 20 30 40 50 60 70 80 90 100
Professionalism ()	

Page Break

© 2021 Xun H et al. *JAMA Network Open*.

Q66 This person walks into your patient room. Based on his attire, what do you think he does?

- Doctor (1)
- Surgeon (2)
- Nurse (3)
- Medical technician (4)
- Physician Assistant (5)

Q68 This person walks into your patient room. Based on his attire, what do you think he does?

- Doctor (1)
- Surgeon (2)
- Nurse (3)
- Medical technician (4)
- Physician Assistant (5)

© 2021 Xun H et al. *JAMA Network Open*.

Q69 This person walks into your patient room. Based on his attire, what do you think he does?

- ○ Doctor (1)
- ○ Surgeon (2)
- ○ Nurse (3)
- ○ Medical technician (4)
- ○ Physician Assistant (5)

Q67 This person walks into your patient room. Based on her attire, what do you think she does?

- ○ Doctor (1)
- ○ Surgeon (2)
- ○ Nurse (3)
- ○ Medical technician (4)
- ○ Physician Assistant (5)

© 2021 Xun H et al. *JAMA Network Open*.

Q70 This person walks into your patient room. Based on her attire, what do you think she does?

- ◯ Doctor (1)
- ◯ Surgeon (2)
- ◯ Nurse (3)
- ◯ Medical technician (4)
- ◯ Physician Assistant (5)

Q71 This person walks into your patient room. Based on her attire, what do you think she does?

- ◯ Doctor (1)
- ◯ Surgeon (2)
- ◯ Nurse (3)
- ◯ Medical technician (4)
- ◯ Physician Assistant (5)

End of Block: GENDER

Start of Block: Demographics

Q2 What is your age?

▼ 18 (1) ... 100 (83)

© 2021 Xun H et al. *JAMA Network Open*.

Q3 What is your gender identity?

- ☐ Male (1)
- ☐ Female (2)
- ☐ Non-binary (5)
- ☐ Other (6) ____

© 2021 Xun H et al. *JAMA Network Open*.

Q4 Please select all the countries below where **you have resided or currently reside.**

- ☐ Afghanistan (1)
- ☐ Albania (2)
- ☐ Algeria (3)
- ☐ Andorra (4)
- ☐ Angola (5)
- ☐ Antigua and Barbuda (6)
- ☐ Argentina (7)
- ☐ Armenia (8)
- ☐ Australia (9)
- ☐ Austria (10)
- ☐ Azerbaijan (11)
- ☐ Bahamas (12)
- ☐ Bahrain (13)
- ☐ Bangladesh (14)
- ☐ Barbados (15)
- ☐ Belarus (16)
- ☐ Belgium (17)

© 2021 Xun H et al. *JAMA Network Open*.

- [ ] Belize (18)
- [ ] Benin (19)
- [ ] Bhutan (20)
- [ ] Bolivia (21)
- [ ] Bosnia and Herzegovina (22)
- [ ] Botswana (23)
- [ ] Brazil (24)
- [ ] Brunei Darussalam (25)
- [ ] Bulgaria (26)
- [ ] Burkina Faso (27)
- [ ] Burundi (28)
- [ ] Cambodia (29)
- [ ] Cameroon (30)
- [ ] Canada (31)
- [ ] Cape Verde (32)
- [ ] Central African Republic (33)
- [ ] Chad (34)
- [ ] Chile (35)

© 2021 Xun H et al. *JAMA Network Open*.

- [ ] China (36)
- [ ] Colombia (37)
- [ ] Comoros (38)
- [ ] Congo, Republic of the... (39)
- [ ] Costa Rica (40)
- [ ] Côte d'Ivoire (41)
- [ ] Croatia (42)
- [ ] Cuba (43)
- [ ] Cyprus (44)
- [ ] Czech Republic (45)
- [ ] Democratic People's Republic of Korea (46)
- [ ] Democratic Republic of the Congo (47)
- [ ] Denmark (48)
- [ ] Djibouti (49)
- [ ] Dominica (50)
- [ ] Dominican Republic (51)
- [ ] Ecuador (52)
- [ ] Egypt (53)

© 2021 Xun H et al. *JAMA Network Open*.

- [ ] El Salvador (54)
- [ ] Equatorial Guinea (55)
- [ ] Eritrea (56)
- [ ] Estonia (57)
- [ ] Ethiopia (58)
- [ ] Fiji (59)
- [ ] Finland (60)
- [ ] France (61)
- [ ] Gabon (62)
- [ ] Gambia (63)
- [ ] Georgia (64)
- [ ] Germany (65)
- [ ] Ghana (66)
- [ ] Greece (67)
- [ ] Grenada (68)
- [ ] Guatemala (69)
- [ ] Guinea (70)
- [ ] Guinea-Bissau (71)

© 2021 Xun H et al. *JAMA Network Open*.

- [ ] Guyana (72)
- [ ] Haiti (73)
- [ ] Honduras (74)
- [ ] Hong Kong (S.A.R.) (75)
- [ ] Hungary (76)
- [ ] Iceland (77)
- [ ] India (78)
- [ ] Indonesia (79)
- [ ] Iran, Islamic Republic of... (80)
- [ ] Iraq (81)
- [ ] Ireland (82)
- [ ] Israel (83)
- [ ] Italy (84)
- [ ] Jamaica (85)
- [ ] Japan (86)
- [ ] Jordan (87)
- [ ] Kazakhstan (88)
- [ ] Kenya (89)

© 2021 Xun H et al. *JAMA Network Open*.

- [ ] Kiribati (90)
- [ ] Kuwait (91)
- [ ] Kyrgyzstan (92)
- [ ] Lao People's Democratic Republic (93)
- [ ] Latvia (94)
- [ ] Lebanon (95)
- [ ] Lesotho (96)
- [ ] Liberia (97)
- [ ] Libyan Arab Jamahiriya (98)
- [ ] Liechtenstein (99)
- [ ] Lithuania (100)
- [ ] Luxembourg (101)
- [ ] Madagascar (102)
- [ ] Malawi (103)
- [ ] Malaysia (104)
- [ ] Maldives (105)
- [ ] Mali (106)
- [ ] Malta (107)

© 2021 Xun H et al. *JAMA Network Open*.

☐ Marshall Islands (108)

☐ Mauritania (109)

☐ Mauritius (110)

☐ Mexico (111)

☐ Micronesia, Federated States of... (112)

☐ Monaco (113)

☐ Mongolia (114)

☐ Montenegro (115)

☐ Morocco (116)

☐ Mozambique (117)

☐ Myanmar (118)

☐ Namibia (119)

☐ Nauru (120)

☐ Nepal (121)

☐ Netherlands (122)

☐ New Zealand (123)

☐ Nicaragua (124)

☐ Niger (125)

© 2021 Xun H et al. *JAMA Network Open*.

- ☐ Nigeria (126)
- ☐ North Korea (127)
- ☐ Norway (128)
- ☐ Oman (129)
- ☐ Pakistan (130)
- ☐ Palau (131)
- ☐ Panama (132)
- ☐ Papua New Guinea (133)
- ☐ Paraguay (134)
- ☐ Peru (135)
- ☐ Philippines (136)
- ☐ Poland (137)
- ☐ Portugal (138)
- ☐ Qatar (139)
- ☐ Republic of Korea (140)
- ☐ Republic of Moldova (141)
- ☐ Romania (142)
- ☐ Russian Federation (143)

© 2021 Xun H et al. *JAMA Network Open*.

☐ Rwanda (144)

☐ Saint Kitts and Nevis (145)

☐ Saint Lucia (146)

☐ Saint Vincent and the Grenadines (147)

☐ Samoa (148)

☐ San Marino (149)

☐ Sao Tome and Principe (150)

☐ Saudi Arabia (151)

☐ Senegal (152)

☐ Serbia (153)

☐ Seychelles (154)

☐ Sierra Leone (155)

☐ Singapore (156)

☐ Slovakia (157)

☐ Slovenia (158)

☐ Solomon Islands (159)

☐ Somalia (160)

☐ South Africa (161)

© 2021 Xun H et al. *JAMA Network Open*.

☐ South Korea (162)

☐ Spain (163)

☐ Sri Lanka (164)

☐ Sudan (165)

☐ Suriname (166)

☐ Swaziland (167)

☐ Sweden (168)

☐ Switzerland (169)

☐ Syrian Arab Republic (170)

☐ Tajikistan (171)

☐ Thailand (172)

☐ The former Yugoslav Republic of Macedonia (173)

☐ Timor-Leste (174)

☐ Togo (175)

☐ Tonga (176)

☐ Trinidad and Tobago (177)

☐ Tunisia (178)

☐ Turkey (179)

© 2021 Xun H et al. *JAMA Network Open*.

- ☐ Turkmenistan (180)
- ☐ Tuvalu (181)
- ☐ Uganda (182)
- ☐ Ukraine (183)
- ☐ United Arab Emirates (184)
- ☐ United Kingdom of Great Britain and Northern Ireland (185)
- ☐ United Republic of Tanzania (186)
- ☐ United States of America (187)
- ☐ Uruguay (188)
- ☐ Uzbekistan (189)
- ☐ Vanuatu (190)
- ☐ Venezuela, Bolivarian Republic of... (191)
- ☐ Viet Nam (192)
- ☐ Yemen (193)
- ☐ Zambia (580)
- ☐ Zimbabwe (1357)
- ☐ Other (1358) ____

© 2021 Xun H et al. *JAMA Network Open*.

© 2021 Xun H et al. *JAMA Network Open*.

Q131 Please select all the countries below where **you have received health care.**

- ▢ Afghanistan (1)
- ▢ Albania (2)
- ▢ Algeria (3)
- ▢ Andorra (4)
- ▢ Angola (5)
- ▢ Antigua and Barbuda (6)
- ▢ Argentina (7)
- ▢ Armenia (8)
- ▢ Australia (9)
- ▢ Austria (10)
- ▢ Azerbaijan (11)
- ▢ Bahamas (12)
- ▢ Bahrain (13)
- ▢ Bangladesh (14)
- ▢ Barbados (15)
- ▢ Belarus (16)
- ▢ Belgium (17)

© 2021 Xun H et al. *JAMA Network Open*.

- ☐ Belize (18)
- ☐ Benin (19)
- ☐ Bhutan (20)
- ☐ Bolivia (21)
- ☐ Bosnia and Herzegovina (22)
- ☐ Botswana (23)
- ☐ Brazil (24)
- ☐ Brunei Darussalam (25)
- ☐ Bulgaria (26)
- ☐ Burkina Faso (27)
- ☐ Burundi (28)
- ☐ Cambodia (29)
- ☐ Cameroon (30)
- ☐ Canada (31)
- ☐ Cape Verde (32)
- ☐ Central African Republic (33)
- ☐ Chad (34)
- ☐ Chile (35)

© 2021 Xun H et al. *JAMA Network Open*.

- [ ] China (36)
- [ ] Colombia (37)
- [ ] Comoros (38)
- [ ] Congo, Republic of the... (39)
- [ ] Costa Rica (40)
- [ ] Côte d'Ivoire (41)
- [ ] Croatia (42)
- [ ] Cuba (43)
- [ ] Cyprus (44)
- [ ] Czech Republic (45)
- [ ] Democratic People's Republic of Korea (46)
- [ ] Democratic Republic of the Congo (47)
- [ ] Denmark (48)
- [ ] Djibouti (49)
- [ ] Dominica (50)
- [ ] Dominican Republic (51)
- [ ] Ecuador (52)
- [ ] Egypt (53)

© 2021 Xun H et al. *JAMA Network Open*.

- [ ] El Salvador (54)
- [ ] Equatorial Guinea (55)
- [ ] Eritrea (56)
- [ ] Estonia (57)
- [ ] Ethiopia (58)
- [ ] Fiji (59)
- [ ] Finland (60)
- [ ] France (61)
- [ ] Gabon (62)
- [ ] Gambia (63)
- [ ] Georgia (64)
- [ ] Germany (65)
- [ ] Ghana (66)
- [ ] Greece (67)
- [ ] Grenada (68)
- [ ] Guatemala (69)
- [ ] Guinea (70)
- [ ] Guinea-Bissau (71)

© 2021 Xun H et al. *JAMA Network Open*.

- ☐ Guyana (72)
- ☐ Haiti (73)
- ☐ Honduras (74)
- ☐ Hong Kong (S.A.R.) (75)
- ☐ Hungary (76)
- ☐ Iceland (77)
- ☐ India (78)
- ☐ Indonesia (79)
- ☐ Iran, Islamic Republic of... (80)
- ☐ Iraq (81)
- ☐ Ireland (82)
- ☐ Israel (83)
- ☐ Italy (84)
- ☐ Jamaica (85)
- ☐ Japan (86)
- ☐ Jordan (87)
- ☐ Kazakhstan (88)
- ☐ Kenya (89)

© 2021 Xun H et al. *JAMA Network Open*.

- ☐ Kiribati (90)
- ☐ Kuwait (91)
- ☐ Kyrgyzstan (92)
- ☐ Lao People's Democratic Republic (93)
- ☐ Latvia (94)
- ☐ Lebanon (95)
- ☐ Lesotho (96)
- ☐ Liberia (97)
- ☐ Libyan Arab Jamahiriya (98)
- ☐ Liechtenstein (99)
- ☐ Lithuania (100)
- ☐ Luxembourg (101)
- ☐ Madagascar (102)
- ☐ Malawi (103)
- ☐ Malaysia (104)
- ☐ Maldives (105)
- ☐ Mali (106)
- ☐ Malta (107)

© 2021 Xun H et al. *JAMA Network Open*.

- [ ] Marshall Islands (108)
- [ ] Mauritania (109)
- [ ] Mauritius (110)
- [ ] Mexico (111)
- [ ] Micronesia, Federated States of... (112)
- [ ] Monaco (113)
- [ ] Mongolia (114)
- [ ] Montenegro (115)
- [ ] Morocco (116)
- [ ] Mozambique (117)
- [ ] Myanmar (118)
- [ ] Namibia (119)
- [ ] Nauru (120)
- [ ] Nepal (121)
- [ ] Netherlands (122)
- [ ] New Zealand (123)
- [ ] Nicaragua (124)
- [ ] Niger (125)

© 2021 Xun H et al. *JAMA Network Open*.

- [ ] Nigeria (126)
- [ ] North Korea (127)
- [ ] Norway (128)
- [ ] Oman (129)
- [ ] Pakistan (130)
- [ ] Palau (131)
- [ ] Panama (132)
- [ ] Papua New Guinea (133)
- [ ] Paraguay (134)
- [ ] Peru (135)
- [ ] Philippines (136)
- [ ] Poland (137)
- [ ] Portugal (138)
- [ ] Qatar (139)
- [ ] Republic of Korea (140)
- [ ] Republic of Moldova (141)
- [ ] Romania (142)
- [ ] Russian Federation (143)

© 2021 Xun H et al. *JAMA Network Open*.

- ☐ Rwanda (144)
- ☐ Saint Kitts and Nevis (145)
- ☐ Saint Lucia (146)
- ☐ Saint Vincent and the Grenadines (147)
- ☐ Samoa (148)
- ☐ San Marino (149)
- ☐ Sao Tome and Principe (150)
- ☐ Saudi Arabia (151)
- ☐ Senegal (152)
- ☐ Serbia (153)
- ☐ Seychelles (154)
- ☐ Sierra Leone (155)
- ☐ Singapore (156)
- ☐ Slovakia (157)
- ☐ Slovenia (158)
- ☐ Solomon Islands (159)
- ☐ Somalia (160)
- ☐ South Africa (161)

© 2021 Xun H et al. *JAMA Network Open*.

- [ ] South Korea (162)
- [ ] Spain (163)
- [ ] Sri Lanka (164)
- [ ] Sudan (165)
- [ ] Suriname (166)
- [ ] Swaziland (167)
- [ ] Sweden (168)
- [ ] Switzerland (169)
- [ ] Syrian Arab Republic (170)
- [ ] Tajikistan (171)
- [ ] Thailand (172)
- [ ] The former Yugoslav Republic of Macedonia (173)
- [ ] Timor-Leste (174)
- [ ] Togo (175)
- [ ] Tonga (176)
- [ ] Trinidad and Tobago (177)
- [ ] Tunisia (178)
- [ ] Turkey (179)

© 2021 Xun H et al. *JAMA Network Open*.

- [ ] Turkmenistan (180)
- [ ] Tuvalu (181)
- [ ] Uganda (182)
- [ ] Ukraine (183)
- [ ] United Arab Emirates (184)
- [ ] United Kingdom of Great Britain and Northern Ireland (185)
- [ ] United Republic of Tanzania (186)
- [ ] United States of America (187)
- [ ] Uruguay (188)
- [ ] Uzbekistan (189)
- [ ] Vanuatu (190)
- [ ] Venezuela, Bolivarian Republic of... (191)
- [ ] Viet Nam (192)
- [ ] Yemen (193)
- [ ] Zambia (580)
- [ ] Zimbabwe (1357)
- [ ] Other (1358) ____

© 2021 Xun H et al. *JAMA Network Open*.

Display This Question:

If List of Countries = United States of America

Q5 In which state in USA did you live in or do you currently reside?

▼ Alabama (1) ... I do not reside in the United States (53)

Q132 Where have you received the majority of your health care?

- ○ Facility in an urban area (1)
- ○ Facility in a suburban area (2)
- ○ Facility in a rural community (3)

Q6 Do you identify as Hispanic or Latino?

- ○ Yes (1)
- ○ No (2)

© 2021 Xun H et al. *JAMA Network Open*.

Q89 Which of the following races do you identify as? (select all that apply)

- ☐ White (1)
- ☐ Black or African American (2)
- ☐ American Indian or Alaska Native (3)
- ☐ Asian (4)
- ☐ Native Hawaiian or Pacific Islander (5)
- ☐ Other (6)
- ☐ Prefer not to answer (7)

Q8 What is the highest level of school you have completed or the highest degree you have received?

- ○ Less than high school degree (1)
- ○ High school graduate (high school diploma or equivalent including GED) (2)
- ○ Some college but no degree (3)
- ○ Associate degree in college (2-year) (4)
- ○ Bachelor's degree in college (4-year) (5)
- ○ Master's degree (6)
- ○ Doctoral/Professional degrees (PhD, JD, MD) (7)

© 2021 Xun H et al. *JAMA Network Open*.

Q9 Approximate household income

▼ (1) ... >200K (7)

Q91 Please set the slider bar to 20.

	0 10 20 30 40 50 60 70 80 90 100
Set slider to 20. ()	

End of Block: Demographics

Start of Block: Present ID

Q91 This is your ID: ${e://Field/Random%20ID}

Please copy this value and paste it into MTurk to receive credit for your work.

YOU MUST HIT NEXT TO SUBMIT YOUR SURVEY - otherwise, you will not be compensated.

Thank you!

End of Block: Present ID

© 2021 Xun H et al. *JAMA Network Open*.

Multivariate Analysis of Respondent Characteristics Correlating with Physician Trait Ratings

PHYSICIAN EXPERIENCE		
**Respondent Characteristics**	**Correlation Coefficient [95% Confidence Interval]**	***P* Value**
Age	0.005 [-0.001, 0.011]	0.085
Female Gender	0.153 [0.046, 0.260]	**0.005**
Education	0.018 [-0.040, 0.076]	0.541
Annual Income	0.022 [-0.024, 0.068]	0.350
Geographic Location: Northeast USA	0.283 [-0.081, 0.647]	0.127
Geographic Location: West USA	0.360 [0.010, 0.710]	**0.044**
Geographic Location: Midwest USA	0.414 [0.045, 0.783]	**0.028**
Geographic Location: South USA	0.338 [-0.006, 0.681]	0.054
Receive care in an urban area	-0.112 [-0.383, 0.159]	0.419
Receive care in a suburban area	0.076 [-0.197, 0.350]	0.583
Been admitted to hospital	0.040 [-0.036, 0.116]	0.301
Had surgery before	-0.030 [-0.180, 0.120]	0.693
Healthcare worker - self	0.212 [0.043, 0.381]	**0.014**
Healthcare worker - family	0.049 [-0.096, 0.194]	0.507
**PHYSICIAN PROFESSIONALISM**		
**Respondent Characteristics**	**Correlation Coefficient [95% Confidence Interval]**	***P* Value**
Age	0.006 [0.001, 0.012]	**0.032**
Female Gender	-0.110 [-0.219, -0.002]	0.047
Education	-0.035 [-0.094, 0.024]	0.248
Annual Income	-0.043 [-0.090, 0.004]	0.074
Geographic Location: Northeast USA	0.105 [-0.265, 0.475]	0.577
Geographic Location: West USA	0.082 [-0.274, 0.438]	0.650
Geographic Location: Midwest USA	-0.098 [-0.473, 0.277]	0.607
Geographic Location: South USA	0.166 [-0.183, 0.515]	0.350
Receive care in an urban area	0.174 [-0.101, 0.450]	0.214
Receive care in a suburban area	-0.067 [-0.345, 0.211]	0.636
Been admitted to hospital	-0.027 [-0.104, 0.050]	0.495
Had surgery before	0.100 [-0.052, 0.252]	0.196
Healthcare worker - self	-0.043 [-0.214, 0.129]	0.625
Healthcare worker - family	0.049 [-0.098, 0.197]	0.510

PHYSICIAN FRIENDLINESS		
**Respondent Characteristics**	**Correlation Coefficient [95% Confidence Interval]**	***P* Value**

© 2021 Xun H et al. *JAMA Network Open*.

Age	-0.011[-0.017, -0.006]	**< 0.0001**
Female Gender	-0.043 [-0.152, -0.066]	0.438
Education	0.017 [-0.042, 0.076]	0.579
Annual Income	0.021 [-0.026, 0.068]	0.383
Geographic Location: Northeast USA	-0.388 [-0.757, -0.019]	0.039
Geographic Location: West USA	-0.442 [-0.797, -0.087]	**0.015**
Geographic Location: Midwest USA	-0.316 [-0.690, 0.058]	0.098
Geographic Location: South USA	-0.504 [-0.852, -0.156]	**0.005**
Receive care in an urban area	-0.063 [-0.338, 0.212]	0.654
Receive care in a suburban area	-0.009 [-0.287, 0.268]	0.946
Been admitted to hospital	-0.013 [-0.090, 0.064]	0.737
Had surgery before	-0.070 [-0.222, 0.082]	0.365
Healthcare worker - self	-0.169 [-0.341, 0.002]	0.052
Healthcare worker - family	-0.099 [-0.246, 0.049]	0.189

eAppendix 2. Multivariate analysis of ranked health care provider values based on respondent characteristics. All respondent characteristics analyzed with ranked healthcare provider ratings of experience, professionalism, and friendliness are reported. Multivariate analysis indicated respondents from the West and Midwest emphasized physician experience (coefficient 0.360, $P < 0.05$, coefficient 0.414, $P < 0.028$ respectively). Older respondent age was positively correlated with emphasis of physician professionalism as a key trait (coefficient 0.006, $P < 0.05$). Younger respondent age was correlated with emphasis of physician friendliness as a key trait (age coefficient -0.011, P value <0.0001).

© 2021 Xun H et al. *JAMA Network Open*.

(A)

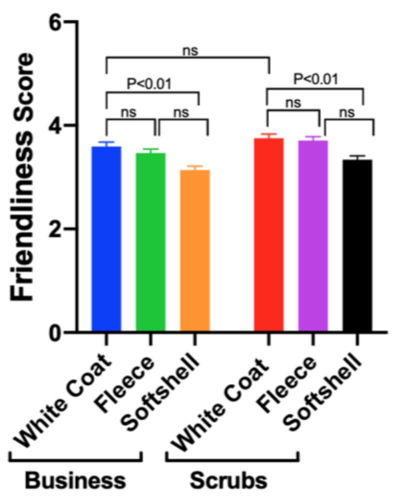

(B)

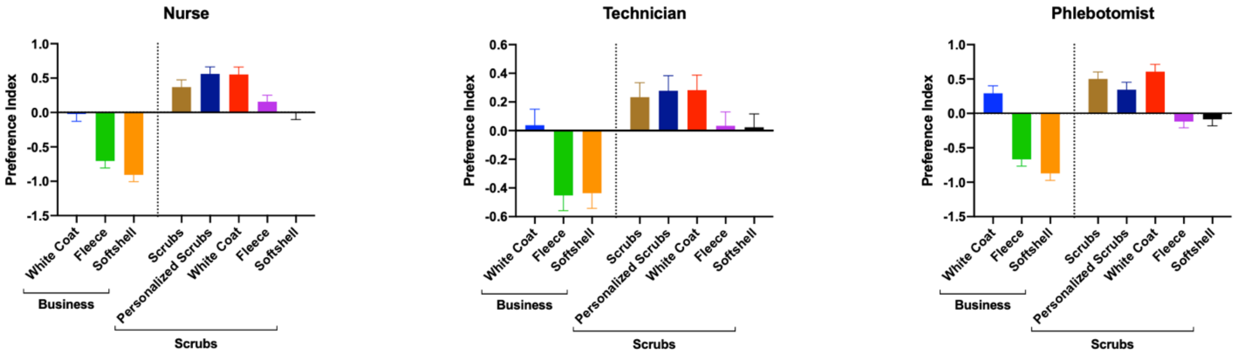

eAppendix 3. Casual physician attire: public ratings of a physician's professionalism, experience, and friendliness by physician attire and preference by profession. (A) Respondents ranked the six physician attires as most friendly to least. *P* values < 0.05 are indicated by brackets. **(B)** Respondent preferences for attire differ by profession, as reported by average preference indices. Respondents were shown photographs of a model wearing business attire innerwear with varying outerwear (left three bars), scrubs innerwear alone (scrubs-scrubs middle bar), or scrubs innerwear with varying outerwear (right four bars). Scrubs refer to unisex hospital grade scrubs; personalized scrubs refer to branded and tailored scrubs, designed to have a more modern fit.

© 2021 Xun H et al. *JAMA Network Open*.

(A)

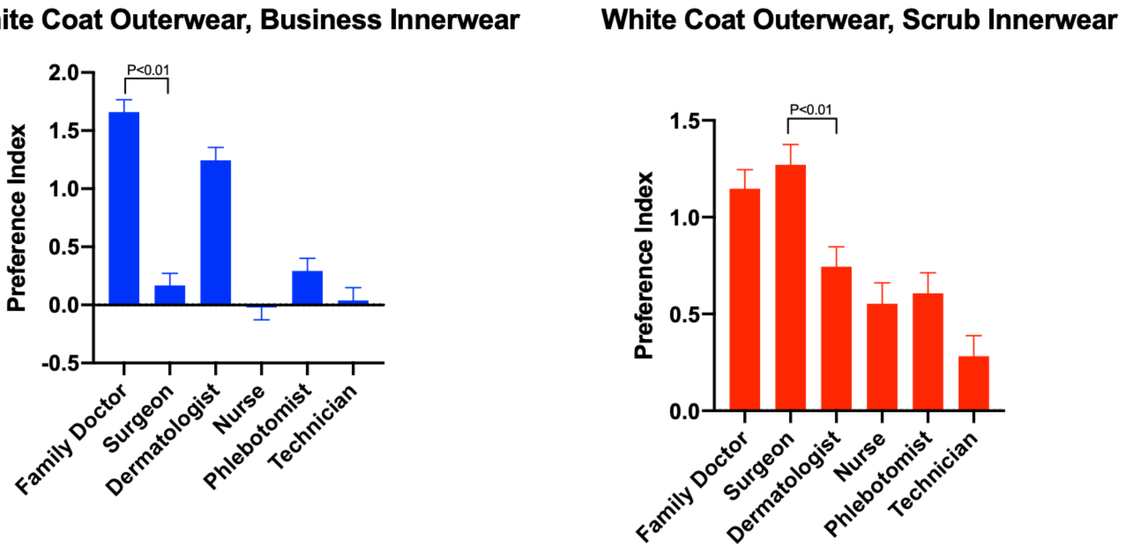

(B)

Multivariate Analysis of Respondent Characteristics Correlating with Physician Ratings by ATTIRE			
**PHYSICIAN EXPERIENCE**			
**Respondent Characteristic**	**Rated Attire**	**Coefficient [95% CI]**	***P* value**
Age	White Coat, Business	0.015 [0.004, 0.026]	0.009
Gender		-0.306 [-0.515, -0.098]	0.004
Healthcare worker - self		0.536 [0.207, 0.864]	0.001
Healthcare worker - self	White Coat, Scrubs	0.514 [0.186, 0.842]	0.002
Had surgery before	Fleece Coat, Business	-0.32 [-0.625, -0.015]	0.040
Healthcare worker - self		-0.427 [-0.770, -0.083]	0.015
Healthcare worker - family		0.314 [0.018, 0.609]	0.037
Age	Fleece Coat, Scrubs	-0.017 [-0.028, -0.006]	0.002
Geographic location: Midwest USA	Softshell Coat, Scrubs	-0.802 [-1.525, -0.078]	0.030
		-0.675 [-1.348, -0.002]	0.049
**PHYSICIAN PROFESSIONALISM**			
**Respondent Characteristic**	**Rated Attire**	**Coefficient [95% CI]**	***P* value**
Age	White Coat, Business	0.015 [0.003, 0.028]	0.016
Healthcare worker - family		0.318 [0.007, 0.629]	0.045
Gender	White Coat, Scrubs	0.21 [0.013, 0.406]	0.036
Education		0.109 [0.002, 0.216]	0.045
Healthcare worker - self		0.47 [0.161, 0.780]	0.003
Age	Fleece Coat, Business	-0.014 [-0.026, -0.003]	0.018

© 2021 Xun H et al. *JAMA Network Open*.

Had surgery before		-0.321 [-0.626, -0.016]	0.039
Geographic location: Northeast USA<br>Geographic location: Midwest USA<br>Geographic location: South USA	Fleece Coat, Scrubs	-0.732 [-1.459, -0.005]	0.049
		-1.092 [-1.830, -0.355]	0.004
		-0.697 [-1.383, -0.011]	0.046
Healthcare worker - self<br>Been admitted to hospital<br>Had surgery before	Softshell Coat, Business	-0.462 [-0.796, -0.128]	0.007
		-0.151[-0.292, -0.009]	0.038
		0.369 [0.088, 0.650]	0.010
Healthcare worker - family	Softshell Coat, Scrubs	-0.313 [-0.585, -0.041]	0.024
**PHYSICIAN FRIENDLINESS**			
**Respondent Characteristic**	**Rated Attire**	**Coefficient [95% CI]**	***P* value**
Receive care in an urban area	White Coat, Business	0.776 [0.090, 1.462]	0.027
Annual Income	Fleece Coat, Business	0.107 [0.004, 0.210]	0.042

eAppendix 4. Public preference of health care provider attire and variables impacting ratings. (A) In particular, while white coat outerwear with business innerwear or scrub innerwear has positive preference indices for multiple professions, they are more strongly preferred for physicians (family doctors, dermatologists, or surgeons). (B) Multivariate analysis of rankings based on respondent characteristics revealed that certain characteristics correlated with higher or lower rankings of experience, professionalism, and friendliness of physician attire. This table summarizes the statistically significant respondent characteristics.

© 2021 Xun H et al. *JAMA Network Open*.

(A)

**Attire**	**Model Gender**	**Rater Characteristic**	**Coefficient [95% CI]**	***P* value**
Outerwear: White Coat<br>Innerwear: Business attire	Male	Healthcare worker - self	5.841 [1.293, 10.388]	**0.012**
		Male Raters from West USA	-21.529 [-27.769, -5.288]	**0.010**
Outerwear: White Coat<br>Innerwear: Scrubs	Male	Geographic location: Midwest USA	11.596 [1.843, 21.349]	**0.020**
		Geographic location: South USA	10.997 [1.923, 20.070]	**0.018**
		Healthcare worker - self	5.532 [1.068, 9.996]	**0.015**
	Female	Gender	3.21 [0.320, 6.099]	**0.030**
		Geographic location: Northeast USA	11.413 [1.557, 21.269]	**0.023**
		Geographic location: Midwest USA	10.591 [0.598, 20.583]	**0.038**
		Geographic location: South USA	10.371 [1.075, 19.668]	**0.029**
		Healthcare worker - self	6.999 [2.425, 11.572]	**0.003**
Outerwear: Softshell<br>Innerwear: Business	Male	Receive care in a suburban area	10.171 [1.135, 19.206]	**0.027**
Outerwear: n/a<br>Innerwear: Fashion Scrubs	Male	Gender	4.097 [0.591, 7.602]	**0.022**
		Annual Income	1.735 [0.216, 3.255]	**0.025**
	Female	Geographic location: South USA	14.333 [1.974, 26.693]	**0.023**
		Gender	3.917 [0.073, 7.761]	**0.046**

(B)

© 2021 Xun H et al. *JAMA Network Open*.

Male Model

Male Raters	White Coat, Business			Fleece Coat, Business			Softshell, Business			White Coat, Scrubs			Fleece Coat, Scrubs			Softshell Coat, Scrubs			Scrubs			Fashion Scrubs		
**Rater Characteristic**	Coefficient	p-value	95% CI	Coefficient	p-value	95% CI	Coefficient	p-value	95% CI	Coefficient	p-value	95% CI	Coefficient	p-value	95% CI	Coefficient	p-value	95% CI	Coefficient	p-value	95% CI	Coefficient	p-value	95% CI
Age	0.202	0.126	-0.057 - 0.461	0.223	0.120	-0.059 - 0.504	0.210	0.185	-0.102 - 0.521	0.187	0.163	-0.077 - 0.451	0.212	0.164	-0.087 - 0.511	0.205	0.177	-0.093 - 0.502	0.005	0.972	-0.270 - 0.280	-0.058	0.710	-0.362 - 0.247
Education	0.489	0.702	-2.030 - 3.008	1.910	0.170	-0.827 - 4.646	0.805	0.601	-2.223 - 3.833	-0.074	0.954	-2.642 - 2.493	-0.628	0.671	-3.534 - 2.279	-0.211	0.886	-3.109 - 2.686	0.119	0.930	-2.552 - 2.790	-0.635	0.673	-3.598 - 2.327
Annual Income	0.628	0.515	-1.273 - 2.529	-0.821	0.434	-2.887 - 1.244	-0.211	0.856	-2.497 - 2.074	0.276	0.779	-1.661 - 2.214	0.215	0.847	-1.979 - 2.409	0.720	0.517	-1.467 - 2.907	0.623	0.543	-1.393 - 2.639	2.154*	0.059	-0.082 - 4.390
Northeast	-9.956	0.244	-26.762 - 6.850	11.617	0.211	-6.643 - 29.877	-2.218	0.829	-22.424 - 17.988	13.443	0.123	-3.687 - 30.573	5.319	0.589	-14.075 - 24.714	7.351	0.454	-11.982 - 26.685	-0.231	0.980	-18.052 - 17.590	6.588	0.512	-13.181 - 26.358
West	-21.529***	0.010	-37.769 - -5.288	1.004	0.911	-16.642 - 18.650	-12.736	0.200	-32.262 - 6.790	2.340	0.781	-14.214 - 18.894	4.673	0.624	-14.069 - 23.415	4.244	0.655	-14.439 - 22.927	-13.229	0.131	-30.450 - 3.992	-4.839	0.618	-23.944 - 14.265
Midwest	-9.291	0.291	-26.592 - 8.011	3.284	0.731	-15.514 - 22.082	-4.455	0.673	-25.256 - 16.346	9.854	0.272	-7.781 - 27.489	2.255	0.824	-17.712 - 22.221	6.427	0.525	-13.477 - 26.330	-9.019	0.334	-27.365 - 9.327	1.089	0.916	-19.264 - 21.441
South	-14.007*	0.090	-30.211 - 2.196	7.886	0.378	-9.720 - 25.492	-6.782	0.493	-26.263 - 12.700	10.193	0.225	-6.323 - 26.710	11.010	0.247	-7.690 - 29.709	14.093	0.138	-4.548 - 32.734	-3.285	0.707	-20.467 - 13.897	13.619	0.160	-5.442 - 32.680
Recieve care in an urban area	-0.866	0.873	-11.539 - 9.807	-5.793	0.326	-17.389 - 5.803	1.943	0.766	-10.889 - 14.774	-1.242	0.822	-12.121 - 9.636	2.163	0.730	-10.154 - 14.480	-1.356	0.828	-13.634 - 10.922	-3.319	0.564	-14.636 - 7.998	-8.349	0.191	-20.903 - 4.206
Recieve care in a suburban area	5.711	0.298	-5.085 - 16.508	-0.429	0.943	-12.159 - 11.301	9.049	0.171	-3.931 - 22.029	3.795	0.497	-7.210 - 14.799	4.153	0.512	-8.306 - 16.612	0.315	0.960	-12.105 - 12.735	0.193	0.974	-11.255 - 11.640	-5.306	0.411	-18.006 - 7.394
Been admitted to hospital	-0.045	0.977	-3.170 - 3.079	0.559	0.746	-2.835 - 3.954	1.185	0.535	-2.571 - 4.941	1.470	0.364	-1.714 - 4.655	0.928	0.612	-2.677 - 4.533	-0.067	0.971	-3.661 - 3.527	0.943	0.575	-2.369 - 4.256	0.981	0.599	-2.694 - 4.656
Had surgery before	-1.863	0.549	-7.985 - 4.258	-4.116	0.224	-10.767 - 2.535	-3.987	0.287	-11.347 - 3.372	-0.804	0.800	-7.043 - 5.436	-5.221	0.147	-12.285 - 1.844	-0.833	0.816	-7.875 - 6.210	-0.803	0.808	-7.294 - 5.688	-2.757	0.451	-9.958 - 4.444
Healthcare worker - self	1.563	0.664	-5.525 - 8.651	-2.511	0.521	-10.212 - 5.190	-3.315	0.444	-11.836 - 5.207	6.371*	0.084	-0.853 - 13.596	0.234	0.955	-7.946 - 8.413	-3.129	0.450	-11.283 - 5.024	-1.085	0.776	-8.600 - 6.431	-1.389	0.743	-9.726 - 6.949
Healthcare worker - family	-0.617	0.830	-6.274 - 5.041	4.199	0.180	-1.948 - 10.346	5.830*	0.093	-0.972 - 12.632	-2.810	0.338	-8.577 - 2.956	0.288	0.931	-6.241 - 6.817	-0.179	0.957	-6.687 - 6.329	-0.814	0.789	-6.813 - 5.185	0.958	0.777	-5.697 - 7.613

Female Raters	White Coat, Business			Fleece Coat, Business			Softshell, Business			White Coat, Scrubs			Fleece Coat, Scrubs			Softshell Coat, Scrubs			Scrubs			Fashion Scrubs		
**Rater Characteristic**	Coefficient	p-value	95% CI	Coefficient	p-value	95% CI	Coefficient	p-value	95% CI	Coefficient	p-value	95% CI	Coefficient	p-value	95% CI	Coefficient	p-value	95% CI	Coefficient	p-value	95% CI	Coefficient	p-value	95% CI
Age	0.093	0.349	-0.102 - 0.289	0.100	0.426	-0.148 - 0.348	-0.025	0.845	-0.279 - 0.229	0.096	0.320	-0.093 - 0.285	0.090	0.494	-0.169 - 0.350	0.029	0.816	-0.215 - 0.273	0.050	0.636	-0.159 - 0.259	0.057	0.651	-0.192 - 0.306
Education	-0.091	0.930	-2.132 - 1.951	0.811	0.538	-1.776 - 3.397	0.334	0.804	-2.315 - 2.983	0.084	0.933	-1.890 - 2.059	1.042	0.449	-1.666 - 3.749	-0.406	0.754	-2.952 - 2.140	-0.012	0.991	-2.190 - 2.165	0.067	0.959	-2.531 - 2.665
Annual Income	1.121	0.187	-0.549 - 2.791	1.034	0.336	-1.081 - 3.150	1.188	0.281	-0.980 - 3.355	0.924	0.261	-0.691 - 2.539	0.414	0.713	-1.801 - 2.629	1.289	0.224	-0.794 - 3.372	0.972	0.283	-0.809 - 2.754	1.256	0.245	-0.869 - 3.382
Northeast	14.241**	0.021	2.193 - 26.290	7.775	0.317	-7.490 - 23.040	6.230	0.433	-9.409 - 21.869	15.564***	0.009	3.910 - 27.218	12.009	0.140	-3.972 - 27.989	-1.044	0.891	-16.073 - 13.985	12.404*	0.058	-0.449 - 25.256	-1.327	0.865	-16.662 - 14.008
West	12.236**	0.040	0.573 - 23.899	5.376	0.474	-9.401 - 20.154	6.277	0.415	-8.861 - 21.416	11.009*	0.056	-0.272 - 22.291	3.647	0.643	-11.823 - 19.116	-8.094	0.274	-22.642 - 6.454	12.243*	0.054	-0.198 - 24.684	2.721	0.718	-12.124 - 17.566
Midwest	14.983**	0.015	2.965 - 27.001	7.868	0.310	-7.359 - 23.095	8.712	0.272	-6.887 - 24.312	12.272**	0.039	0.647 - 23.897	6.235	0.442	-9.705 - 22.175	-6.172	0.418	-21.163 - 8.819	13.820**	0.035	1.000 - 26.640	11.395	0.144	-3.902 - 26.691
South	14.290**	0.012	3.114 - 25.466	3.304	0.646	-10.856 - 17.464	4.249	0.565	-10.258 - 18.756	10.146*	0.066	-0.665 - 20.957	4.664	0.536	-10.159 - 19.488	-6.128	0.387	-20.069 - 7.813	10.153*	0.095	-1.769 - 22.075	6.910	0.340	-7.315 - 21.135
Recieve care in an urban area	3.657	0.478	-6.489 - 13.803	7.181	0.272	-5.674 - 20.037	11.058*	0.099	-2.112 - 24.227	-2.413	0.629	-12.227 - 7.402	7.192	0.294	-6.265 - 20.649	8.314	0.197	-4.342 - 20.970	1.752	0.750	-9.071 - 12.575	-3.528	0.591	-16.442 - 9.386
Recieve care in a suburban area	1.908	0.712	-8.250 - 12.065	6.635	0.311	-6.234 - 19.505	13.748**	0.041	0.564 - 26.933	1.810	0.717	-8.016 - 11.635	7.026	0.305	-6.447 - 20.498	7.595	0.239	-5.076 - 20.265	3.639	0.509	-7.196 - 14.475	-1.939	0.768	-14.867 - 10.990
Been admitted to hospital	0.713	0.608	-2.024 - 3.450	0.383	0.828	-3.085 - 3.851	-0.500	0.782	-4.053 - 3.053	1.836	0.173	-0.812 - 4.483	-0.172	0.926	-3.802 - 3.459	0.534	0.758	-2.880 - 3.949	-0.499	0.736	-3.419 - 2.420	-1.232	0.487	-4.716 - 2.252
Had surgery before	-2.359	0.392	-7.772 - 3.054	0.176	0.960	-6.683 - 7.034	-2.464	0.490	-9.490 - 4.562	3.508	0.188	-1.728 - 8.744	5.581	0.127	-1.598 - 12.761	5.421	0.115	-1.331 - 12.173	2.914	0.321	-2.860 - 8.688	4.983	0.156	-1.906 - 11.873
Healthcare worker - self	9.239***	0.003	3.257 - 15.220	-0.410	0.915	-7.989 - 7.168	0.777	0.844	-6.987 - 8.541	4.580	0.120	-1.206 - 10.366	-1.774	0.660	-9.707 - 6.160	-1.915	0.614	-9.376 - 5.546	6.264*	0.054	-0.116 - 12.645	1.507	0.697	-6.106 - 9.120
Healthcare worker - family	3.450	0.218	-2.056 - 8.956	-0.018	0.996	-6.994 - 6.958	4.527	0.213	-2.619 - 11.674	5.044*	0.063	-0.282 - 10.369	-3.156	0.395	-10.459 - 4.146	-0.183	0.958	-7.051 - 6.685	3.480	0.244	-2.393 - 9.353	3.576	0.316	-3.432 - 10.584

All Raters	White Coat, Business			Fleece Coat, Business			Softshell, Business			White Coat, Scrubs			Fleece Coat, Scrubs			Softshell Coat, Scrubs			Scrubs			Fashion Scrubs		
**Rater Characteristic**	Coefficient	p-value	95% CI	Coefficient	p-value	95% CI	Coefficient	p-value	95% CI	Coefficient	p-value	95% CI	Coefficient	p-value	95% CI	Coefficient	p-value	95% CI	Coefficient	p-value	95% CI	Coefficient	p-value	95% CI
Age	0.142*	0.074	-0.014 - 0.297	0.126	0.175	-0.056 - 0.309	0.060	0.537	-0.131 - 0.251	0.124	0.110	-0.028 - 0.277	0.100	0.305	-0.091 - 0.291	0.073	0.437	-0.111 - 0.257	0.016	0.846	-0.148 - 0.181	-0.014	0.889	-0.203 - 0.176
Gender	1.290	0.380	-1.595 - 4.175	-1.464	0.396	-4.849 - 1.922	-0.832	0.645	-4.382 - 2.718	2.617*	0.069	-0.207 - 5.441	-0.093	0.959	-3.646 - 3.460	0.855	0.624	-2.567 - 4.278	1.818	0.242	-1.233 - 4.869	4.097**	0.022	0.591 - 7.602
Education	-0.177	0.824	-1.744 - 1.390	1.145	0.222	-0.694 - 2.983	0.311	0.752	-1.616 - 2.237	-0.043	0.957	-1.581 - 1.495	0.413	0.674	-1.516 - 2.341	-0.107	0.910	-1.965 - 1.751	-0.386	0.648	-2.044 - 1.273	-0.524	0.591	-2.437 - 1.389
Annual Income	1.138*	0.073	-0.106 - 2.383	0.297	0.689	-1.163 - 1.758	0.736	0.345	-0.795 - 2.266	0.750	0.228	-0.471 - 1.972	0.434	0.578	-1.098 - 1.965	1.088	0.148	-0.388 - 2.563	1.045	0.120	-0.272 - 2.362	1.735**	0.025	0.216 - 3.255
Northeast	7.336	0.142	-2.464 - 17.136	10.722*	0.068	-0.777 - 22.222	4.284	0.485	-7.767 - 16.335	15.135***	0.002	5.516 - 24.755	8.975	0.144	-3.085 - 21.035	1.621	0.784	-9.999 - 13.241	9.316*	0.078	-1.054 - 19.687	2.573	0.673	-9.392 - 14.538
West	-0.104	0.983	-9.531 - 9.324	4.186	0.458	-6.876 - 15.248	-1.121	0.849	-12.714 - 10.471	6.698	0.156	-2.556 - 15.952	3.982	0.500	-7.619 - 15.584	-3.974	0.485	-15.152 - 7.204	2.165	0.670	-7.811 - 12.141	-1.379	0.814	-12.889 - 10.131
Midwest	7.952	0.116	-1.984 - 17.888	7.618	0.200	-4.041 - 19.278	5.178	0.405	-7.040 - 17.397	11.596**	0.020	1.843 - 21.349	4.841	0.437	-7.387 - 17.069	-1.117	0.852	-12.899 - 10.664	6.198	0.247	-4.317 - 16.712	6.919	0.263	-5.212 - 19.050
South	5.626	0.232	-3.618 - 14.870	6.864	0.214	-3.982 - 17.711	1.408	0.808	-9.959 - 12.775	10.997**	0.018	1.923 - 20.070	7.830	0.177	-3.546 - 19.205	2.146	0.701	-8.815 - 13.106	6.939	0.164	-2.843 - 16.721	10.350*	0.072	-0.936 - 21.636
Recieve care in an urban area	1.281	0.730	-6.011 - 8.573	0.651	0.881	-7.906 - 9.207	5.993	0.190	-2.974 - 14.961	-2.348	0.520	-9.506 - 4.810	4.324	0.344	-4.650 - 13.298	2.999	0.496	-5.648 - 11.646	-0.615	0.876	-8.332 - 7.101	-5.344	0.239	-14.247 - 3.559
Recieve care in a suburban area	2.929	0.434	-4.418 - 10.277	2.233	0.611	-6.388 - 10.855	10.171**	0.027	1.135 - 19.206	2.297	0.532	-4.915 - 9.509	5.079	0.270	-3.962 - 14.121	3.740	0.399	-4.972 - 12.452	1.673	0.673	-6.102 - 9.447	-3.290	0.471	-12.260 - 5.680
Been admitted to hospital	0.349	0.737	-1.689 - 2.387	0.327	0.788	-2.064 - 2.718	0.185	0.884	-2.320 - 2.691	1.632	0.109	-0.368 - 3.633	0.253	0.843	-2.255 - 2.761	0.113	0.927	-2.303 - 2.530	0.195	0.859	-1.962 - 2.351	-0.063	0.960	-2.551 - 2.425
Had surgery before	-1.851	0.366	-5.875 - 2.172	-1.208	0.615	-5.929 - 3.514	-2.851	0.258	-7.799 - 2.097	1.175	0.559	-2.775 - 5.125	1.012	0.688	-3.940 - 5.963	2.533	0.297	-2.238 - 7.304	0.565	0.795	-3.693 - 4.822	0.521	0.835	-4.392 - 5.433
Healthcare worker - self	5.841**	0.012	1.293 - 10.388	-0.888	0.744	-6.224 - 4.448	-0.568	0.842	-6.161 - 5.024	5.532**	0.015	1.068 - 9.996	-0.425	0.882	-6.021 - 5.172	-2.328	0.397	-7.720 - 3.064	3.452	0.159	-1.360 - 8.264	0.722	0.798	-4.830 - 6.274
Healthcare worker - family	0.910	0.647	-2.988 - 4.808	1.373	0.556	-3.201 - 5.947	4.510*	0.065	-0.284 - 9.303	0.906	0.642	-2.920 - 4.732	-1.803	0.461	-6.600 - 2.994	-0.589	0.802	-5.211 - 4.033	0.481	0.819	-3.644 - 4.606	1.396	0.565	-3.363 - 6.155

Female Model

Male Raters	White Coat, Business			Fleece Coat, Business			Softshell, Business			White Coat, Scrubs			Fleece Coat, Scrubs			Softshell Coat, Scrubs			Scrubs			Fashion Scrubs		
**Rater Characteristic**	Coefficient	p-value	95% CI	Coefficient	p-value	95% CI	Coefficient	p-value	95% CI	Coefficient	p-value	95% CI	Coefficient	p-value	95% CI	Coefficient	p-value	95% CI	Coefficient	p-value	95% CI	Coefficient	p-value	95% CI
Age	-0.207	0.179	-0.511 - 0.096	0.072	0.663	-0.253 - 0.397	-0.067	0.675	-0.380 - 0.246	0.172	0.216	-0.101 - 0.444	0.172	0.275	-0.138 - 0.481	0.256	0.103	-0.052 - 0.563	0.129	0.379	-0.160 - 0.419	-0.039	0.816	-0.366 - 0.289
Education	-0.037	0.980	-2.986 - 2.913	0.661	0.680	-2.498 - 3.819	-0.010	0.995	-3.055 - 3.036	0.373	0.781	-2.277 - 3.024	-0.527	0.730	-3.534 - 2.481	0.286	0.851	-2.708 - 3.279	0.941	0.510	-1.872 - 3.755	-0.334	0.836	-3.519 - 2.850
Annual Income	-0.074	0.948	-2.300 - 2.152	-0.123	0.919	-2.507 - 2.261	0.372	0.750	-1.926 - 2.671	0.889	0.382	-1.112 - 2.890	-0.018	0.988	-2.288 - 2.252	0.659	0.566	-1.600 - 2.918	0.043	0.968	-2.080 - 2.167	1.755	0.151	-0.648 - 4.159
Northeast	-8.105	0.418	-27.786 - 11.576	12.044	0.261	-9.033 - 33.121	11.558	0.263	-8.762 - 31.878	16.993*	0.060	-0.694 - 34.679	6.186	0.544	-13.883 - 26.255	13.255	0.192	-6.718 - 33.229	6.044	0.526	-12.731 - 24.818	13.337	0.217	-7.912 - 34.585
West	-14.960	0.123	-33.979 - 4.059	4.989	0.630	-15.379 - 25.357	5.181	0.604	-14.455 - 24.818	6.828	0.432	-10.264 - 23.920	3.523	0.721	-15.871 - 22.917	10.925	0.266	-8.376 - 30.227	-6.089	0.509	-24.231 - 12.054	2.026	0.846	-18.507 - 22.560
Midwest	-4.105	0.690	-24.366 - 16.157	6.348	0.565	-15.350 - 28.047	9.275	0.383	-11.644 - 30.194	15.478*	0.095	-2.730 - 33.686	1.197	0.909	-19.464 - 21.858	14.004	0.181	-6.558 - 34.566	-3.740	0.703	-23.067 - 15.588	-1.339	0.904	-23.214 - 20.536
South	-6.077	0.528	-25.053 - 12.898	11.496	0.266	-8.826 - 31.818	14.437	0.148	-5.155 - 34.029	17.803**	0.041	0.751 - 34.856	10.594	0.282	-8.755 - 29.944	19.319**	0.049	0.062 - 38.577	5.510	0.549	-12.591 - 23.611	14.926	0.152	-5.561 - 35.413
Recieve care in an urban area	7.361	0.247	-5.137 - 19.860	-7.565	0.266	-20.950 - 5.820	-1.724	0.793	-14.629 - 11.180	-4.145	0.468	-15.377 - 7.086	-0.533	0.934	-13.278 - 12.212	-3.529	0.584	-16.213 - 9.155	-5.527	0.362	-17.449 - 6.396	-7.535	0.272	-21.029 - 5.959
Recieve care in a suburban area	8.794	0.172	-3.849 - 21.437	-6.315	0.359	-19.854 - 7.225	-2.120	0.749	-15.173 - 10.934	-1.318	0.819	-12.680 - 10.044	0.226	0.972	-12.666 - 13.118	-0.911	0.889	-13.742 - 11.919	-1.362	0.824	-13.423 - 10.698	-4.183	0.546	-17.833 - 9.467
Been admitted to hospital	-2.096	0.260	-5.754 - 1.563	0.451	0.821	-3.467 - 4.369	-1.119	0.560	-4.896 - 2.659	1.417	0.397	-1.871 - 4.705	0.520	0.784	-3.211 - 4.251	1.193	0.527	-2.520 - 4.906	1.251	0.480	-2.239 - 4.741	-1.175	0.558	-5.125 - 2.775
Had surgery before	-6.293*	0.085	-13.462 - 0.875	-6.102	0.119	-13.779 - 1.575	-6.821*	0.071	-14.222 - 0.581	0.986	0.763	-5.456 - 7.428	-6.096	0.102	-13.406 - 1.214	-0.448	0.904	-7.723 - 6.827	-2.188	0.529	-9.026 - 4.650	-3.885	0.324	-11.625 - 3.855
Healthcare worker - self	-1.607	0.703	-9.907 - 6.693	-5.769	0.202	-14.658 - 3.120	-6.411	0.142	-14.980 - 2.159	8.362**	0.028	0.902 - 15.821	-0.313	0.942	-8.776 - 8.151	3.045	0.477	-5.379 - 11.468	0.034	0.993	-7.884 - 7.952	-7.040	0.123	-16.001 - 1.922
Healthcare worker - family	0.519	0.877	-6.107 - 7.144	6.113*	0.091	-0.982 - 13.209	7.570**	0.030	0.730 - 14.411	-4.336	0.153	-10.290 - 1.618	0.304	0.929	-6.452 - 7.060	-0.499	0.884	-7.223 - 6.224	-2.833	0.378	-9.153 - 3.487	4.901	0.178	-2.252 - 12.054

Female Raters	White Coat, Business			Fleece Coat, Business			Softshell, Business			White Coat, Scrubs			Fleece Coat, Scrubs			Softshell Coat, Scrubs			Scrubs			Fashion Scrubs		
**Rater Characteristic**	Coefficient	p-value	95% CI	Coefficient	p-value	95% CI	Coefficient	p-value	95% CI	Coefficient	p-value	95% CI	Coefficient	p-value	95% CI	Coefficient	p-value	95% CI	Coefficient	p-value	95% CI	Coefficient	p-value	95% CI
Age	-0.178	0.207	-0.455 - 0.099	-0.006	0.964	-0.280 - 0.268	-0.119	0.401	-0.397 - 0.159	0.093	0.342	-0.099 - 0.285	-0.055	0.672	-0.309 - 0.199	0.031	0.802	-0.212 - 0.274	-0.030	0.807	-0.270 - 0.211	-0.013	0.925	-0.292 - 0.265
Education	1.451	0.323	-1.437 - 4.339	2.282	0.117	-0.574 - 5.139	0.469	0.750	-2.431 - 3.370	0.379	0.710	-1.626 - 2.384	0.802	0.551	-1.847 - 3.451	0.696	0.589	-1.838 - 3.230	1.315	0.303	-1.193 - 3.823	0.132	0.929	-2.771 - 3.035
Annual Income	1.402	0.244	-0.960 - 3.765	0.203	0.864	-2.134 - 2.540	1.117	0.355	-1.256 - 3.490	0.594	0.476	-1.046 - 2.234	-0.593	0.590	-2.761 - 1.574	0.353	0.737	-1.720 - 2.426	0.626	0.549	-1.426 - 2.678	0.316	0.793	-2.059 - 2.691
Northeast	3.150	0.716	-13.898 - 20.198	-5.111	0.551	-21.973 - 11.751	0.031	0.997	-17.092 - 17.153	8.626	0.152	-3.209 - 20.461	0.538	0.946	-15.099 - 16.175	4.310	0.571	-10.647 - 19.267	2.973	0.693	-11.832 - 17.778	5.596	0.521	-11.539 - 22.730
West	7.867	0.349	-8.635 - 24.370	1.659	0.841	-14.664 - 17.982	1.752	0.835	-14.823 - 18.327	5.529	0.343	-5.927 - 16.986	-4.602	0.550	-19.739 - 10.536	-0.170	0.982	-14.648 - 14.309	2.747	0.706	-11.585 - 17.078	10.428	0.217	-6.159 - 27.014
Midwest	-0.503	0.954	-17.508 - 16.502	-4.168	0.626	-20.988 - 12.651	-1.573	0.856	-18.652 - 15.507	8.782	0.144	-3.023 - 20.588	-3.567	0.653	-19.165 - 12.031	3.615	0.634	-11.305 - 18.534	-0.617	0.934	-15.385 - 14.151	11.099	0.202	-5.993 - 28.190
South	6.865	0.393	-8.949 - 22.678	0.225	0.977	-15.416 - 15.866	3.366	0.677	-12.517 - 19.248	6.090	0.276	-4.888 - 17.068	-7.378	0.317	-21.884 - 7.127	0.668	0.925	-13.206 - 14.542	-0.721	0.918	-14.455 - 13.012	11.951	0.140	-3.944 - 27.845
Recieve care in an urban area	1.128	0.877	-13.228 - 15.484	2.787	0.699	-11.413 - 16.986	6.674	0.363	-7.745 - 21.093	-3.499	0.490	-13.466 - 6.467	5.989	0.371	-7.180 - 19.157	7.114	0.267	-5.482 - 19.709	-1.561	0.805	-14.029 - 10.907	-5.731	0.435	-20.160 - 8.698
Recieve care in a suburban area	7.280	0.319	-7.092 - 21.653	4.744	0.512	-9.472 - 18.959	6.137	0.403	-8.298 - 20.572	2.806	0.580	-7.171 - 12.784	7.338	0.274	-5.845 - 20.521	7.583	0.237	-5.026 - 20.193	3.714	0.558	-8.768 - 16.196	-5.555	0.449	-20.001 - 8.890
Been admitted to hospital	1.111	0.572	-2.762 - 4.984	0.417	0.831	-3.414 - 4.247	0.375	0.850	-3.515 - 4.265	1.093	0.424	-1.596 - 3.781	-0.077	0.966	-3.630 - 3.475	-0.593	0.731	-3.991 - 2.805	0.893	0.602	-2.471 - 4.256	-0.708	0.720	-4.601 - 3.185
Had surgery before	0.060	0.988	-7.600 - 7.719	0.208	0.957	-7.368 - 7.783	2.416	0.537	-5.277 - 10.109	4.099	0.130	-1.218 - 9.416	3.678	0.303	-3.348 - 10.703	8.047**	0.019	1.327 - 14.767	6.641*	0.050	-0.011 - 13.292	4.409	0.260	-3.289 - 12.107
Healthcare worker - self	3.002	0.485	-5.462 - 11.465	-1.898	0.656	-10.269 - 6.474	-3.103	0.473	-11.604 - 5.397	5.712*	0.057	-0.163 - 11.588	-0.785	0.842	-8.548 - 6.978	0.807	0.831	-6.619 - 8.232	3.686	0.324	-3.664 - 11.036	1.304	0.763	-7.203 - 9.810
Healthcare worker - family	-0.694	0.861	-8.484 - 7.097	-4.286	0.274	-11.992 - 3.419	-2.538	0.523	-10.363 - 5.286	1.959	0.476	-3.449 - 7.367	-3.262	0.369	-10.408 - 3.884	-0.811	0.815	-7.646 - 6.024	-0.199	0.954	-6.964 - 6.567	3.399	0.393	-4.431 - 11.229

All Raters	White Coat, Business			Fleece Coat, Business			Softshell, Business			White Coat, Scrubs			Fleece Coat, Scrubs			Softshell Coat, Scrubs			Scrubs			Fashion Scrubs		
**Rater Characteristic**	Coefficient	p-value	95% CI	Coefficient	p-value	95% CI	Coefficient	p-value	95% CI	Coefficient	p-value	95% CI	Coefficient	p-value	95% CI	Coefficient	p-value	95% CI	Coefficient	p-value	95% CI	Coefficient	p-value	95% CI
Age	-0.177*	0.084	-0.378 - 0.024	0.010	0.926	-0.195 - 0.215	-0.110	0.286	-0.313 - 0.092	0.111	0.163	-0.045 - 0.267	0.001	0.992	-0.191 - 0.194	0.091	0.339	-0.096 - 0.278	0.008	0.929	-0.173 - 0.190	-0.048	0.649	-0.256 - 0.160
Gender	-0.689	0.716	-4.417 - 3.038	-2.797	0.149	-6.598 - 1.004	-0.909	0.635	-4.673 - 2.855	3.210**	0.030	0.320 - 6.099	-0.859	0.637	-4.435 - 2.718	2.038	0.249	-1.429 - 5.506	1.629	0.343	-1.743 - 5.000	3.917**	0.046	0.073 - 7.761
Education	0.865	0.401	-1.158 - 2.888	1.574	0.135	-0.493 - 3.641	0.399	0.702	-1.645 - 2.442	0.340	0.672	-1.236 - 1.916	0.381	0.700	-1.561 - 2.322	0.684	0.476	-1.201 - 2.568	0.989	0.289	-0.843 - 2.820	-0.103	0.923	-2.199 - 1.992
Annual Income	0.744	0.363	-0.863 - 2.351	-0.012	0.989	-1.654 - 1.630	0.692	0.403	-0.931 - 2.315	0.794	0.213	-0.458 - 2.045	-0.233	0.767	-1.775 - 1.309	0.581	0.446	-0.915 - 2.078	0.513	0.489	-0.942 - 1.968	1.019	0.229	-0.645 - 2.683
Northeast	0.057	0.993	-12.595 - 12.709	2.285	0.729	-10.644 - 15.215	4.983	0.444	-7.796 - 17.763	11.413**	0.023	1.557 - 21.269	2.087	0.736	-10.055 - 14.229	6.558	0.275	-5.227 - 18.343	5.286	0.365	-6.170 - 16.742	9.853	0.140	-3.250 - 22.956
West	-1.146	0.853	-13.316 - 11.025	2.091	0.741	-10.347 - 14.528	2.645	0.673	-9.649 - 14.938	3.991	0.409	-5.490 - 13.472	-2.202	0.711	-13.882 - 9.478	2.498	0.665	-8.839 - 13.834	-1.708	0.761	-12.728 - 9.313	6.740	0.294	-5.865 - 19.345
Midwest	-0.020	0.998	-12.848 - 12.807	0.495	0.941	-12.613 - 13.604	3.594	0.586	-9.363 - 16.551	10.591**	0.038	0.598 - 20.583	-1.859	0.767	-14.170 - 10.451	6.904	0.257	-5.044 - 18.853	-0.975	0.869	-12.590 - 10.640	6.580	0.331	-6.706 - 19.865
South	3.420	0.574	-8.514 - 15.354	4.794	0.440	-7.401 - 16.989	8.343	0.174	-3.711 - 20.397	10.371**	0.029	1.075 - 19.668	0.031	0.996	-11.422 - 11.483	7.491	0.186	-3.625 - 18.607	3.211	0.560	-7.595 - 14.017	14.333**	0.023	1.974 - 26.693
Recieve care in an urban area	4.217	0.379	-5.198 - 13.631	-2.489	0.611	-12.110 - 7.132	2.821	0.560	-6.689 - 12.330	-4.342	0.245	-11.676 - 2.992	2.431	0.597	-6.604 - 11.466	1.034	0.817	-7.735 - 9.803	-3.753	0.387	-12.278 - 4.771	-5.111	0.304	-14.861 - 4.640
Recieve care in a suburban area	6.950	0.151	-2.535 - 16.436	-1.785	0.718	-11.478 - 7.909	1.555	0.750	-8.026 - 11.136	0.832	0.825	-6.557 - 8.221	3.284	0.479	-5.819 - 12.387	3.086	0.493	-5.749 - 11.922	0.632	0.885	-7.957 - 9.221	-4.022	0.421	-13.846 - 5.801
Been admitted to hospital	-0.343	0.798	-2.974 - 2.288	0.484	0.723	-2.204 - 3.173	-0.404	0.765	-3.062 - 2.253	1.157	0.268	-0.892 - 3.207	0.054	0.967	-2.471 - 2.578	0.161	0.897	-2.290 - 2.611	0.875	0.471	-1.507 - 3.257	-0.816	0.557	-3.540 - 1.909
Had surgery before	-2.596	0.327	-7.790 - 2.599	-1.871	0.489	-7.179 - 3.438	-1.368	0.609	-6.615 - 3.879	2.053	0.319	-1.993 - 6.100	-0.676	0.790	-5.661 - 4.309	3.992	0.106	-0.846 - 8.831	2.223	0.353	-2.480 - 6.927	-0.266	0.923	-5.646 - 5.114
Healthcare worker - self	0.900	0.763	-4.971 - 6.770	-3.311	0.279	-9.310 - 2.689	-4.430	0.143	-10.360 - 1.500	6.999***	0.003	2.425 - 11.572	0.105	0.971	-5.529 - 5.739	1.935	0.487	-3.534 - 7.403	2.696	0.319	-2.620 - 8.012	-2.004	0.517	-8.085 - 4.076
Healthcare worker - family	-0.094	0.971	-5.127 - 4.938	0.089	0.973	-5.053 - 5.232	2.095	0.419	-2.989 - 7.178	-1.385	0.488	-5.305 - 2.535	-2.214	0.368	-7.043 - 2.616	-1.102	0.644	-5.789 - 3.586	-2.360	0.309	-6.917 - 2.196	3.267	0.219	-1.945 - 8.479

eAppendix 5. Multivariate analysis of professionalism ratings for male and female model stratified by all raters, only male raters, and only female raters. (A) Statistically significant rater characteristics that result in statistically significant differences of professionalism scores for male or female models on multivariate analysis. (B) All rater characteristics that result in no statistically significant ratings.

© 2021 Xun H et al. *JAMA Network Open*.

	n (%) *
**White Coat**	
Outpatient Clinic	302 (62.0)
Inpatient Hospital Wards	323 (66.3)
Emergency Room or Urgent Care	283 (58.1)
Surgical Area	161 (33.1)
**Scrubs**	
Outpatient Clinic	188 (38.6)
Inpatient Hospital Wards	281 (57.7)
Emergency Room or Urgent Care	354 (72.7)
Surgical Area	322 (66.1)
**Fleece Jacket or Vest**	
Outpatient Clinic	331 (68.0)
Inpatient Hospital Wards	165 (33.9)
Emergency Room or Urgent Care	126 (25.9)
Surgical Area	49 (10.1)
**Softshell Jacket**	
Outpatient Clinic	312 (64.1)
Inpatient Hospital Wards	157 (32.2)
Emergency Room/Urgent Care	139 (28.5)
Surgical Area	61 (12.5)

eTable 1. Respondent reported health care locations where the specific physician attire (white coat, scrubs, fleece jacket or vest, and softshell jacket) has been previously seen.

© 2021 Xun H et al. *JAMA Network Open*.

	Male (%)	Female (%)	*P* value	Chi-square, df (*P* value)
**White Coat Business Attire**				51.42, 4
Doctor	430 (88.3)	349 (71.7)	**<0.0001**	(***P* < 0.0001**)
Surgeon	20 (4.1)	24 (4.9)	0.54	
Nurse	10 (2.1)	19 (3.9)	0.09	
Medical Tech	16 (3.3)	39 (8.0)	**<0.005**	
Physician Assistant	11 (2.3)	56 (11.5)	**<0.0001**	
**Scrubs**				12.81, 4
Doctor	23 (4.7)	13 (2.7)	0.09	(***P* = 0.0123**)
Surgeon	241 (49.5)	198 (40.7)	**0.01**	
Nurse	133 (27.3)	161 (33.1)	**0.05**	
Medical Tech	66 (13.6%)	82 (16.8)	0.15	
Physician Assistant	24 (4.9)	33 (6.8)	0.22	
**Fleece Jackets, Scrubs**				7.566, 4
Doctor	20 (4.1)	10 (2.1)	0.06	(***P* = 0.1088**)
Surgeon	10 (2.1)	13 (2.7)	0.53	
Nurse	83 (17.0)	105 (21.6)	0.07	
Medical Tech	231 (47.4)	209 (42.9)	0.16	
Physician Assistant	143 (29.4)	150 (30.8)	0.63	

eTable 2. Respondents' collated votes and percentages and *P* values between male vs female models.

© 2021 Xun H et al. *JAMA Network Open*.
